# Study of bat diversity (Mammalia, Chiroptera) in Xuan Nha Nature Reserve, Son La Province, north-western Vietnam, based on integrative insights from morphology, genetics and echolocation data

**DOI:** 10.3897/BDJ.13.e165516

**Published:** 2025-11-04

**Authors:** Yen H Vu, Hai T Bui, Toan T Giang, Luong K Vu, Luong T Nguyen, Masaharu Motokawa, Son T Nguyen

**Affiliations:** 1 University of Science, Vietnam National University (VNU), Hanoi, Vietnam University of Science, Vietnam National University (VNU) Hanoi Vietnam; 2 Institute of Biology, Vietnam Academy of Science and Technology (VAST), Hanoi, Vietnam Institute of Biology, Vietnam Academy of Science and Technology (VAST) Hanoi Vietnam; 3 Vietnam Nation University of Forestry, Hanoi, Vietnam Vietnam Nation University of Forestry Hanoi Vietnam; 4 The Kyoto University Museum, Kyoto University, Kyoto, Japan The Kyoto University Museum, Kyoto University Kyoto Japan; 5 Graduate University of Science and Technology, Vietnam Academy of Science and Technology (VAST), Hanoi, Vietnam Graduate University of Science and Technology, Vietnam Academy of Science and Technology (VAST) Hanoi Vietnam

**Keywords:** biodiversity, Chiroptera, cytochrome b, species richness

## Abstract

This study presents the results of bat diversity surveys in Xuan Nha Nature Reserve, north-western Vietnam. A total of 114 individuals, representing 19 species belonging to four families, were recorded. The Rhinolophidae family was the most species-rich, contributing eight species to the total diversity, followed by Hipposideridae, with four species. Eight species, including *Rhinolophus episcopus*, *R*.* siamensis*, *R*. cf.* episcopus*, *R*.* perniger*, *Hipposideros griffini*, *Megaerops niphanae*, *Tylonycteris tonkinensis* and *Myotis muricola*, were newly recorded for Xuan Nha NR. Species richness was high, but evenness was low, with *Hipposideros poutensis* and *Rhinolophus pearsonii* dominating captures. Eleven species were observed, represented by only one individual. The morphological examinations, with support from echolocation calls and preliminary genetic analysis, revealed the presence of morphologically conserved and potentially cryptic taxa. Reproductive data indicated early wet-season breeding for several species. Compared to previous surveys in the region, our study substantially expands the knowledge of chiropteran fauna in Xuan Nha. Given the high proportion of habitat-specialist and montane-associated species and the documented presence of a conservation-priority taxon, *Hipposideros griffin*, in Xuan Nha NR, continued biodiversity surveys incorporating molecular and acoustic methods are essential to refine species inventories and to provide information for conservation strategies for this biologically important landscape.

## Introduction

Vietnam, located in Indochina, is recognised as a hotspot country and amongst the world's richest regions for mammal diversity. Such biodiversity is attributed to the country's complex topography, diverse climatic conditions and distinct ecosystems (Sterling et al. 2006, Tordoff et al. 2012). Northern Vietnam, in particular, with numerous protected areas, serves as important refugia for both widespread and endemic bat species. Xuan Nha Nature Reserve (NR), in Xuan Nha Commune of Son La Province includes both primary and secondary evergreen forests, with limestone areas interspersed amongst them (The People’s Committee of Son La Province 2019). Regarding bat diversity, Nguyen et al. (2012) recorded a total of 19 species at this area; nevertheless, data on environmental conditions, as well as detailed morphological characteristics and genetic information of the recorded species, remain unavailable.

In August 2024, within the framework of the Nagao NEF Project, we conducted an 11-day field survey in Xuan Nha NR. The results significantly expand current knowledge of bat diversity in the region by increasing the number of recorded species and providing understandings of undocumented taxa. This paper aims to update the chiropteran fauna of Xuan Nha NR by providing information related to morphological characteristics, echolocation calls and genetic information; and then we discuss species composition and diversity in north-western Vietnam to support future ecological and taxonomic research in the region.

## Material and methods

### Study area

**Figure 1.**
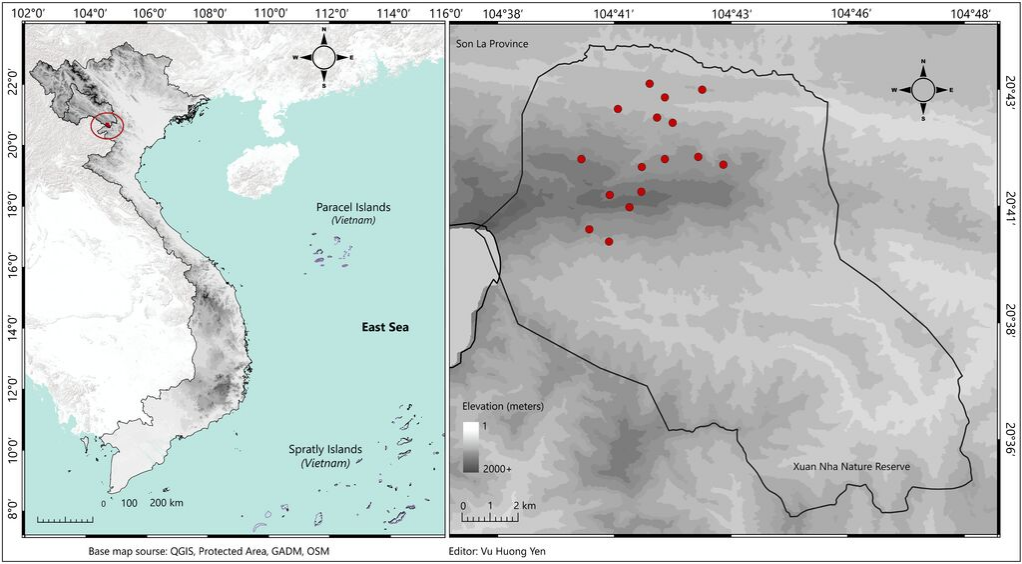
Map of Vietnam showing the location of Xuan Nha NR within Son La Province. Red dots indicate bat sampling sites. The map was organised using QGIS 3.38.3 (https://www.qgis.org).

Xuan Nha NR (20°36'–20°48'N, 104°29'–104°50'E) is positioned in the south-western area of Son La Province, north-western Vietnam, bordering Laos to the west (Fig. 1) and is one of four nature reserves in Son La (The People’s Committee of Son La Province 2019). The Reserve is dominated by tropical forests and interspersed with substantial areas of limestone forest ecosystems (Tordoff et al. 2004). Pha Luong Mountain (1970 m a.s.l.) is the highest peak and located on the mountainous ridge that delineates the Vietnam–Laos border, serving as an important ecological corridor for montane fauna (Center for Nature Conservation and Development (CEV) 2005).

**Figure 2.**
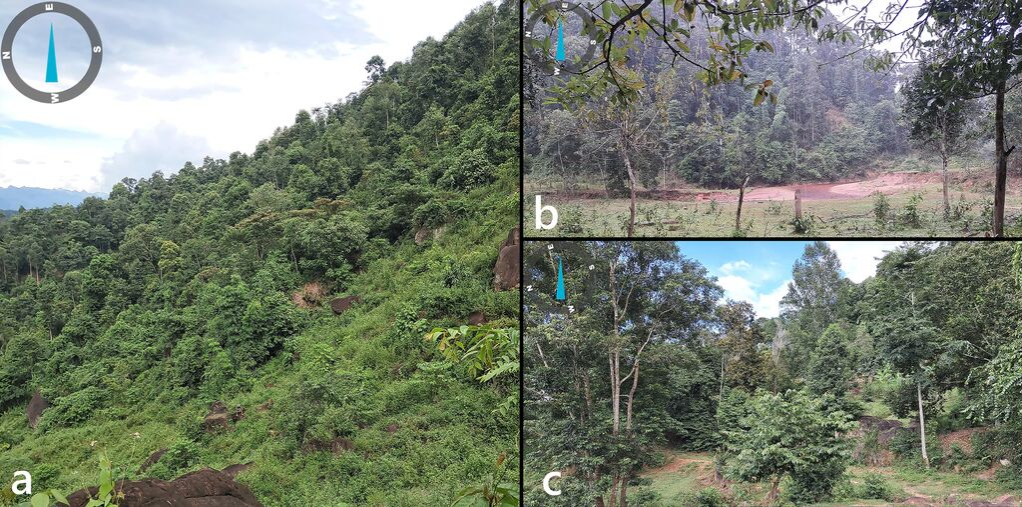
Habitat natures in Xuan Nha NR. **(a)** Tropical evergreen forest at 1000 m a.s.l.; **(b)** Mixed habitat including forest edges and open areas at around 600 m a.s.l.; **(c)** Disturbed secondary forest at 900 m a.s.l.

Along the mid-elevation transects (500–800 m a.s.l.), the surveyed routes crossed areas heavily altered by anthropogenic activity. Remnant patches of primary forest were fragmented and embedded in a matrix of cultivated lands and secondary vegetation (Fig. 2c). Dense thickets of low-trunk tracts predominate between 700 and 900 m a.s.l., while limestone outcrops was observed near a bat cave at approx. 910 m a.s.l. Above 1000 m a.s.l., undisturbed forest patches persist along the slopes (Fig. 2a). These forests have a canopy height of 20–30 m with a dense understory of saplings, climbing vines and dense shrubs. At elevations above 1500 m a.s.l., nearly pristine montane moss forests remain, characterised by high canopy trees (20–50 m), abundant epiphytes and closed canopy cover. The region has cooler temperatures and high humidity. Streams formed by precipitation patterns and seasonal runoff are typically 1.5–4.5 m wide and less than 0.7 m deep.

### Survey methods and sampling

**Figure 3.**
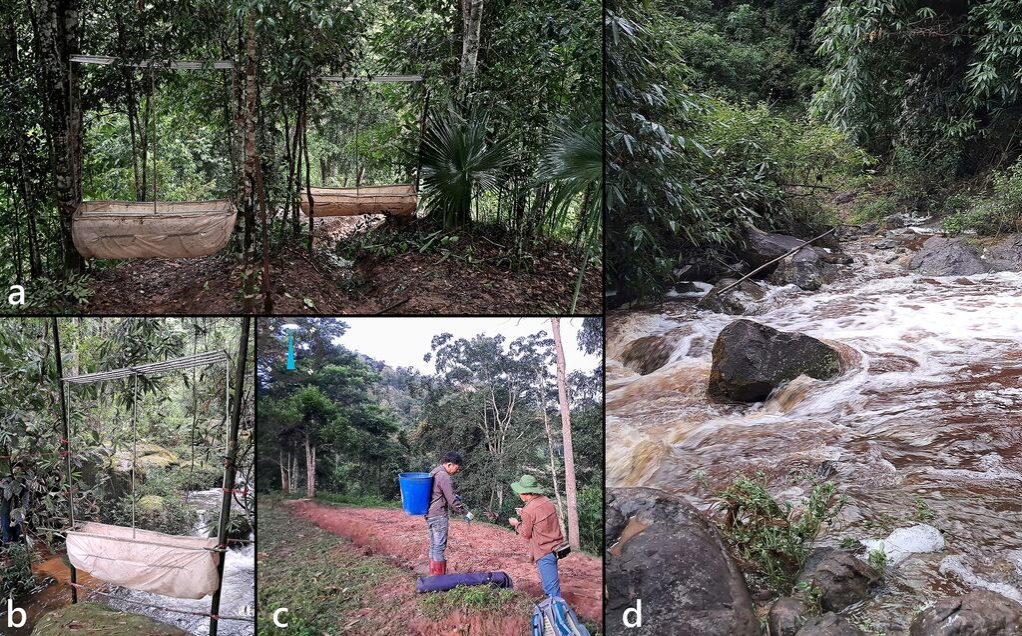
Bat trapping deployment: **(a, b, d)** Double-harp traps set along forest trails and over streams; **(c)** Setup of mist nets.

The main field survey was conducted from 4-15 August 2024, with additional data from a two-day survey on 25-26 March 2010. Bats were surveyed using mist nets and harp traps (Tidemann and Woodside 1978) set along four elevational bands (ca. 600, 700, 900 and 1100 m a.s.l.) (Fig. 1). Sixteen trapping sites were established, each operated for 1–2 nights depending on weather and accessibility. Harp traps (1.8 m high × 1.2 m wide) with four parallel rows of monofilament lines (2.5 cm spacing) were deployed at each site (Tidemann and Woodside 1978). Traps were strategically placed along bat flyways such as: forest trails, ridgelines, streams and cave entrances (Fig. 3a, b and d). At least one harp trap was installed per site and set before dusk, remaining open until midnight or later depending on bat activities. Traps were checked every 30–45 minutes, synchronised with mist net inspections, to minimise stress and injury to captured individuals. Mist nets were deployed at ground level across presumed flyways. Two net sizes were used: 9 × 3 m and 12 × 3 m, constructed from black nylon with four shelves (Fig. 3c). Nets were mounted on fiberglass or in combinations with locally sourced bamboo poles. Sampling was conducted from 18:00 h to 23:00 h and 04:00 h to 05:00 h, with 1–3 nets operated per site per night. Nets were closed during the day to avoid accidental captures of birds or livestock. Geographic coordinates and elevations were recorded using the Gaia GPS (WGS84 datum).

### Echolocation recordings and analyses

Echolocation calls were recorded using an Echo Meter Touch 2 ultrasonic detector (Wildlife Acoustics, Inc., Maynard, Massachusetts, USA) connected to the smartphone device. Recordings were obtained under two conditions: (1) handheld recordings made immediately at the mist net site of captured individuals; (2) controlled recordings inside a temporary flight tent (4 × 4 × 2 m). All recordings were analysed visually using spectrograms to characterise call structure and frequency parameters. Echolocation calls were compared with references from previous studies in Vietnam (Nguyen et al. 2021, Győrössy et al. 2024) to assist species identification, particularly for cryptic taxa or species difficult to capture.

### Specimen examination

Most specimens (109 individuals) were collected during the main survey in 2024, while only five individuals were captured on March 2010, with detailed information for each specimen presented in Suppl. material 1. Photographs of live specimens were taken using a Canon EOS Kiss X7 digital camera with an EF-S 18–55 mm f/3.5–5.6 kit lens. Standard morphometric measurements were followed Wilson and Mittermeier (2019). The reproductive condition of females was assessed following Racey (2009) and age class was determined by the degree of phalangeal epiphyseal fusion (Brunet-Rossinni and Wilkinson 2009). Pregnancy in females was checked by gentle abdominal palpation.

**Figure 4.**
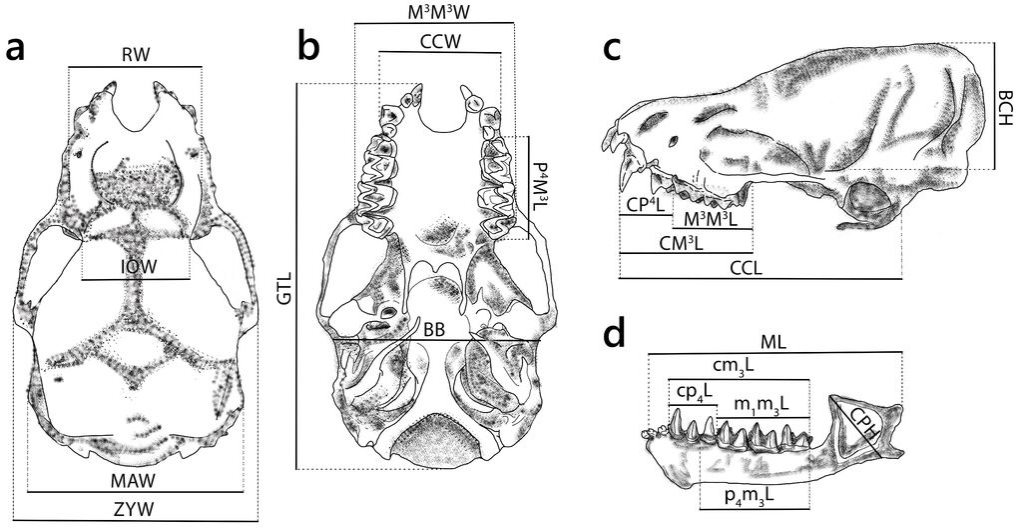
**(a) **Dorsal, **(b) **ventral, **(c)** lateral views of the cranium; **(d)** lateral views of mandible displaying craniodental measurements. The diagram is constructed, based on the skull morphology of *P*. *tenuis *species.

Specimens were preserved in 95% ethanol during both the 11-days survey in 2024 and 2-days in 2010. Upon transfer to the laboratory, ethanol concentration was reduced to 70% for long-term storage. Skulls were extracted and cleaned and one to two individuals per species were selected for genetic analyses. Craniodental characters were measured under a stereoscopic microscope (SMZ 745, Nikon) using an electronic digital caliper (Mitutoyo NTD12-15PMX, 0.01 mm precision). A total of 20 metrics were measured following Vu et al. (2024) (Fig. 4; Suppl. material 2). Specimens were identified, based on external and cranial morphology, supplemented by DNA analysis and echolocation data, using comparative references (Borissenko and Kruskop 2003, Vu 2012, Kruskop 2013, Vuong et al. 2015, Vuong et al. 2017a, Vuong et al. 2017b, Vuong et al. 2018, Moratelli et al. 2019, Vu et al. 2024). Voucher specimens and associated tissue samples are currently deposited in the Department of Zoology, Institute of Biology (IB), VAST, Hanoi, Vietnam.

### Molecular data and phylogenetic analyses

DNA was isolated from tissue samples preserved in 99% ethanol using the DNeasy® Blood & Tissue Kit (Qiagen, Hilden, Germany). The mitochondrial cytochrome b (Cyt *b*) gene was targeted for species-level identification. PCR amplification employed primer pair SoriF/SoriR (Bui et al. 2020a) with the following thermal cycling conditions: initial denaturation at 95°C for 5 min; 35 cycles of 95°C for 30 s, 55°C for 50 s and 72°C for 2 min; and a final extension at 72°C for 10 min. PCR products were purified and sequenced by 1st BASE (Selangor, Malaysia) using Sanger sequencing.

Chromatograms were edited and assembled in Chromas Pro (Technelysium Pty Ltd., Australia) and MEGA 11 (Tamura et al. 2021). Sequences were aligned with MUSCLE in MEGA 11. The final alignment of the Cyt *b* fragment was 1140 bp. Phylogenetic analyses were performed using Maximum Likelihood (ML) under the GTR+G+I model, selected by the Bayesian Information Criterion (BIC) in ModelFinder implementation in IQ-TREE v.1.6.12 (Nguyen et al. 2015). Node support was evaluated with 10,000 ultrafast bootstrap replicates (Hoang et al. 2018). The resulting phylogenetic trees were visualised using FigTree v.1.4.4 and edited using Adobe Photoshop 2023.

## Data resources

**Data package title:** Occurrence dataset of bats (Mammalia, Chiroptera) from Xuan Nha Nature Reserve, Son La Province, north-western Vietnam.

**Resource link:** https://doi.org/10.15468/7d54s5

**Number of datasets:** 1

**Data set name:** Occurrence dataset of bats (Mammalia, Chiroptera) from Xuan Nha Nature Reserve, Son La Province, north-western Vietnam.

**Data format:** Darwin Core Event

**Description:** This dataset presents species occurrence of bats collected from Xuan Nha NR, Son La Province, north-western Vietnam. Field surveys were conducted in August 2024 as part of the biodiversity research programme with the support of the Nagao Natural Environment Foundation (NEF). For every occurrence record, the dataset provides information on location (GPS coordinates), date, sex, reproductive condition and trap type.

## Results

### Species richness, diversity and distribution

A total of 114 bat individuals were collected during two field surveys in Xuan Nha NR, representing 19 species from nine genera and four families: Hipposideridae, Pteropodidae, Rhinolophidae and Vespertilionidae. The Rhinolophidae was the most species-rich family, with eight species of one genus (*R*. *pearsonii*, *R*. *perniger*,* R*.* affinis*, *R*.* pusillus*, *R*.* episcopus*, *R*.* siamensis*, *R*.* thomasi* and *R*. cf.* episcopus*). This was followed by Hipposideridae, with four species of two genera (*A*.* stoliczkanus*, *H*.* armiger*, *H*.* griffini* and *H*.* poutensis*) and Vespertilionidae, with five species of four genera (*K*. cf. *dongduongana*, *M*.* alticraniatus*, *M*.* muricola*, *P*.* tenuis* and *T*.* tonkinensis*). The Pteropodidae family was represented by two species of two genera (*C*.* sphinx* and *M*.* niphanae*) (Table 1). *H*.* poutensis* was the most abundant species, with 39 individuals (34.2%). The second most common was *R*.* pearsonii* (32 individuals, 28.1%). Other frequently captured species included *R*.* thomasi* (7), *C*.* sphinx* (8) and *M*.* niphanae* (7). In contrast, eleven species were represented by a single individual, including *R*.* pusillus*, *R*.* affinis*, *R*.* episcopus*, *R*.* siamensis*, *R*. cf.* episcopus*, *R*.* perniger*, *H*.* griffini*, *M*.* alticraniatus*, *M*.* muricola*, *T*.* tonkinensis* and *T*.* tenuis*. 

Capture success varied by trap type: harp traps accounted for 88 individuals (77.2%), proving effective for *Rhinolophus* and *Hipposideros* species, while mist nets (26 individuals, 22.8%) exclusively captured frugivorous taxa, such as *C*.* sphinx* and *M*.* niphanae*. Species evenness was low, with captures dominated by a few abundant taxa. Signs of reproductive activity, including pregnancy, lactation or the presence of newborns, were observed in 15 females across five species, while 54 females showed no evidence of breeding.

### Chiropteran species recorded from Xuan Nha NR

#### Family Hipposideridae

Four species of Hipposideridae were recorded (*H*.* armiger*, *H*.* griffini*, *H*.* poutensis*, *A*. *stoliczkanus*). *H*.* griffini* is the first record from Xuan Nha NR. *H*.* larvatus* in the previous report from this area (Nguyen et al. 2012) have since been reclassified as *H*.* poutensis* following Yuzefovich et al. (2022). *H*.* pomona*, previously reported in the region, are reclassified as *H*.* gentillis *(Srinivasulu and Srinivasulu 2018), but was not confirmed in this study.

***Hipposideros armiger*** **(Hodgson, 1835)**

**Figure 5.**
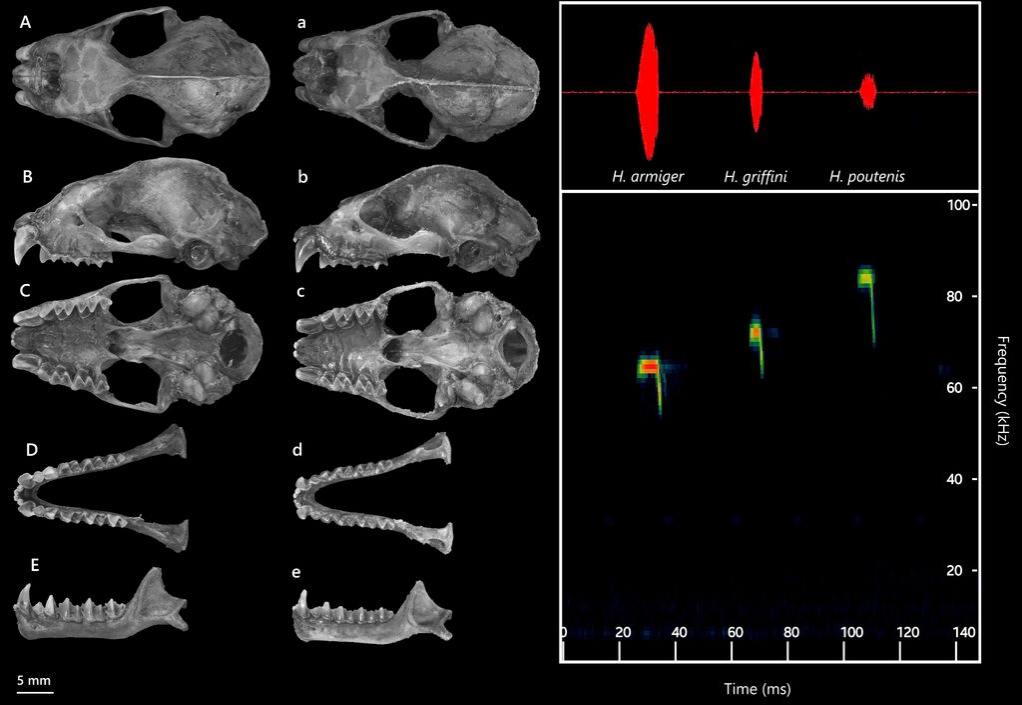
**(Left)** Dorsal (A, a), lateral (B, b), ventral (C, c) views of the cranium and dorsal (D, d), lateral (E, e) views of the mandible, comparing two closely-related species: *H*.* armiger *and *H*.* griffini*; **(Right)** Ultrasonic echolocation calls of Hipposideridae bats.

*H*.* armiger* is amongst the largest members of Hipposideridae and widely distributed across Southeast Asia (Kruskop 2013). Three individuals were collected, none of the specimens showing signs of reproductive activity. External measurements included HB 87.7–95.7 mm, FA 92.6–98.9 mm and Wt 47–66 g. Dorsal pelage ranged from dark grey-brown (in one female individual) to black fur (two males), with grey ventral fur. A pair of swollen structures was observed above each eye, posterior to the nose-leaf, but these were less developed than in *H*.* griffini* individuals. Cranially (Fig. 5), *H*. *armiger* shows a large, robust skull, with GTL averaging 32.1 mm and CCL 30.7 mm. The sagittal crest is prominent. The rostrum and supraorbital regions are inflated, giving a distinct cranial profile compared to *H*.* griffini*. Dentition is characterised by large upper canines (C^1^) closely aligned with P^4^, a reduced and laterally displaced P^2^ and shallow frontal depressions in lateral view.

The echolocation call of a male *H*. *armiger* was recorded at the capture site (mist net, 650 m a.s.l.). This individual emitted a narrowband CF-FM structure (Fig. 5, right). The CF component had a peak frequency of 63.6 ± 0.7 kHz (mean ± SD), with a maximum start frequency of 65.8 kHz and a minimum end frequency of 55.7 kHz. The mean call duration was 9.1 ± 0.3 ms, with the longest call lasting 10.2 ms.

***Hipposideros griffini*** **Vu, Puechmaille, Denzinger, Dietz, Csorba, Bates, Teeling & Schnitzler, 2012**

*H*. *griffini *was first described from Cat Ba Island and subsequently recorded in Chu Mom Ray NP (Central Highlands) and Cat Tien NP (southern part of Vietnam) (Vu 2012, Vu et al. 2012, Vu 2015, Vu 2019). In this study, an adult male was captured at approx. 650 m a.s.l. in disturbed secondary forest, together with *H*. *armiger*. The external measurements of male were as follows: HB 88.8 mm, T 60.3 mm, HF 16.3 mm, E 28.3 mm, FA 87.6 mm and Wt 54 g (Table 2). This individual has dark grey-black dorsal fur, with paler plumage ventrally. The male also possesses well-developed fleshy outgrowths and prominent glandular sacs behind the posterior nose-leaf.

**Figure 6.**
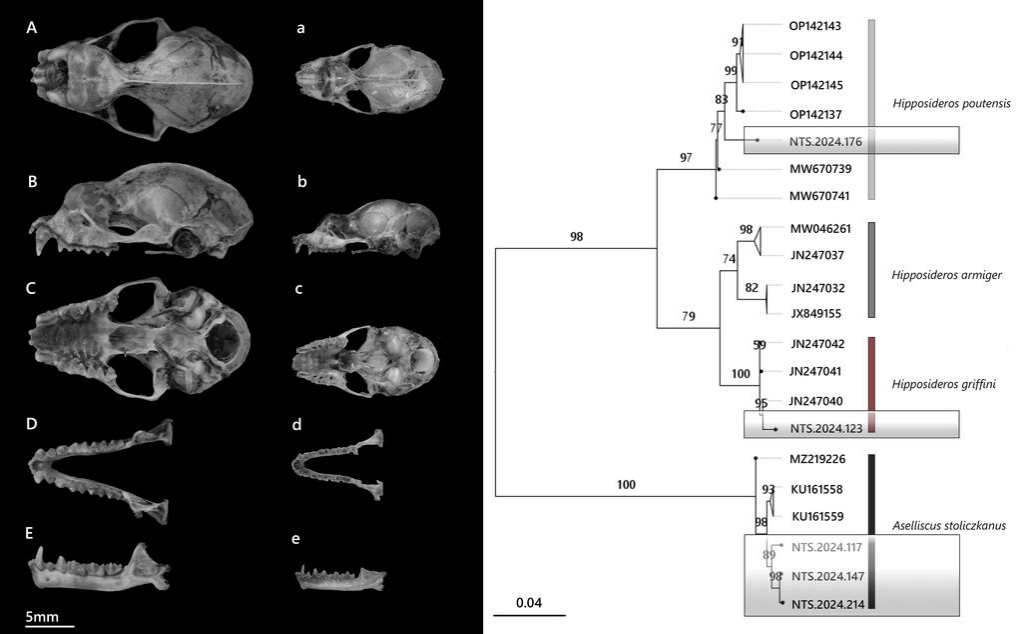
(**Left**) Cranium of *H*. *poutensis* (A–C) and *A*. *stoliczkanus* (a–c) in dorsal (A, a), lateral (B, b) and ventral (C, c) views; mandible in dorsal (D, d) and lateral (E, e) views; (**Right**) Phylogeny based on Cyt *b* sequences of Hipposideridae bats. Bootstrap support values (BS) are shown at nodes.

The skull is robust, with a smooth dorsal cranial profile. The braincase is flattened and less globular, with a short, flattened rostrum lacking the steep slope seen in *H*.* armiger*. The rostrum–frontal transition forms a gentle incline, the sagittal crest is well developed and the lambda point is prominent. Zygomatic arches are strong and evenly curved, without the inward bend typical of *H*.* armiger* (Fig. 5, left). The upper dentition is massive with molars bearing distinct cusps. The mandible is significantly smaller than in *H*. *armiger*. The coronoid process is straight and triangular rather than posteriorly curved. Lower teeth are massive and closely set, but smaller than in *H*.* armiger* (Table 3). The specimen clustered with previously published sequences of *H*. *griffini* (JN247040–JN247042), forming a well-supported monophyletic group (Fig. 6, right), with pairwise genetic divergence of approximately 0.9% from other *H*.* griffini* populations.

The echolocation call of an adult male consisted of narrowband CF-FM signals (Fig. 5, right). The CF had a peak frequency of 74.7 ± 0.5 kHz, with start and end frequencies of 73.8 kHz and 63.4 kHz, respectively. The mean call duration was 7.2 ± 0.3 ms, with the longest call lasting 7.3 ms.

***Hipposideros poutensis*** **Allen, 1906**

**Figure 7.**
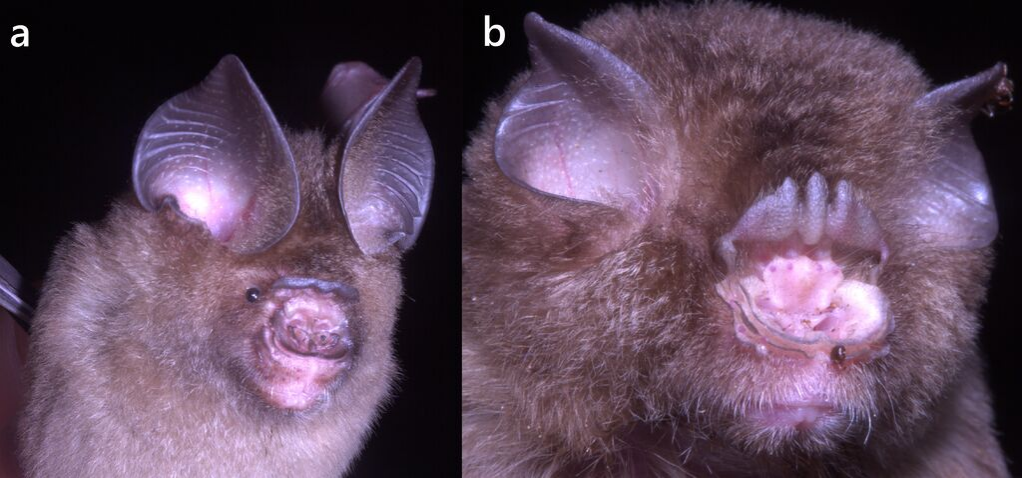
Selected Hipposideridae species recorded on Xuan Nha NR: **(a**)**** *H*.* poutensis*; **(**b)**** *A*.* stolickzanus*.

In the study, 39 individuals of *H*.* poutensis* (21♂, 18♀) were recorded (Table 1, Suppl. material 1). Amongst females, only three showed active reproductive status as lactating, while the remaining 15 were non-reproductive. *H*.* poutensis* is a medium-sized leaf-nosed bat (Fig. 7a), characterised by a slender skull compared to *H*.* armiger* or *H*.* griffini*, with a developed sagittal crest, a narrow braincase and a slightly swollen rostrum.

The specimen clustered within the *H*.* poutensis* lineage on the phylogenetic tree, with strong support and showed the closest genetic relationship with specimens collected from Bai Tu Long Island (OP142137), followed by population from Cat Ba Island (OP142143–OP142145) (Fig. 6, right). Pairwise genetic divergence ranged between 2% and 3.6%. These findings support the recent taxonomic distinction of *H*.* poutensis* from *H*.* larvatus* as reported by Yuzefovich et al. (2022), with both morphological and molecular analyses confirming its status as the distinct species. Based on recordings of a single male individual, this species emitted narrowband CF-FM calls (Fig. 5, right) with a peak frequency of 85.6 ± 0.7 kHz, starting at 88.7 kHz and ending at 70.7 kHz. Mean call duration was 6.2 ± 0.2 ms, with the longest call lasting 6.5 ms.

***Aselliscus stoliczkanus*** **(Dobson, 1871)**

*A*.* stoliczkanus* is widely distributed across north-western and central Vietnam (Vuong et al. 2015). In this study, three specimens were identified as *A*.* stoliczkanus*, based on diagnostic external and cranial traits. These bats are small-sized, with a tricuspid posterior nose-leaf, short bodies and bicoloured dorsal fur (white bases and brown tips), while the ventral fur is paler (Fig. 7b). Cranial features include a slightly swollen rostrum and a relatively elongated snout compared to members of Hipposideridae (Fig. 6, left). The newly-obtained specimens clustered with reference *A. stolickzanus* sequences (BS = 89–98%) (Fig. 6, right). Genetic divergence between the study specimens and known references ranged from 0.5% to 1.5%.

#### Family Pteropodidae

Two species of fruit bats were recorded during this survey (Table 1). *C*.* sphinx*, previously reported from the Reserve, was re-confirmed, while *M*. *niphanae* represents a new record for this locality. Both species exhibited signs of reproductive activity during August.

***Cynopterus sphinx*** **(Vahl, 1797)**

**Figure 8.**
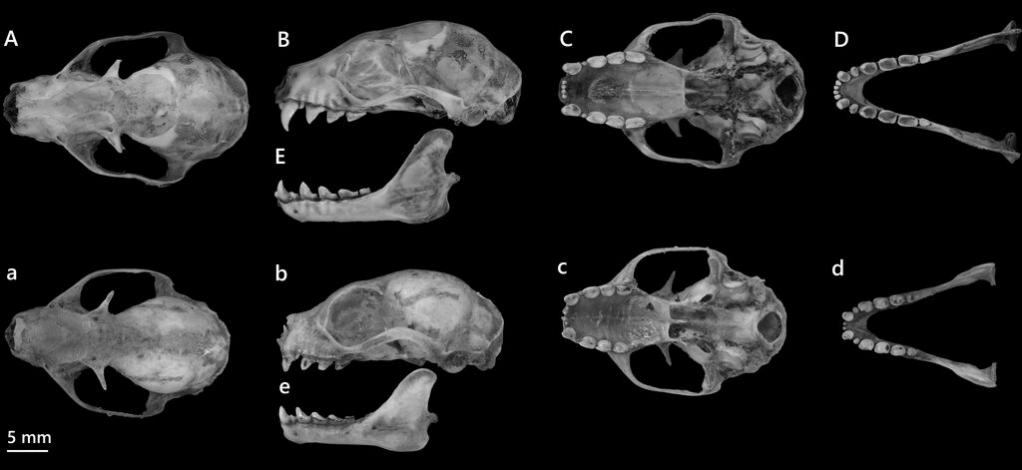
Dorsal (A, a), lateral (B, b), ventral (C, c) views of the cranium and Dorsal (D, d), lateral (E, e) views of the mandible of *C*.* sphinx* and *M*.* niphanae*, respectively.

*C*. *sphinx* is common, occurring across lowland and edge habitats. Adults show short orange-brown dorsal pelage, a greyish ventral side and a darker mantle region, more pronounced in males. Juveniles are paler with an overall greyish tone. The ears are brown with a distinctive whitish margin and the interfemoral membrane is narrow, but evident, with a short tail extending slightly beyond it. Compared with *Megaerops*, *C*.* sphinx* is larger-bodied, more robust, with a more developed interfemoral membrane and a diagnostic white ear margin (absent in *Megaerops*). Cranially, the skull is elongate, narrowing anteriorly (Fig. 8), with expanded zygomatic arches and a broad, elongated palate. The dentition consists of robust molars with slightly rounded cusps and elongated upper canines.

***Megaerops niphanae*** **Yenbutra & Felten, 1983**

This is a small pteropodid bat. The species is easily recognised by its soft, light brownish-grey pelage and absence of a tail. The flight membranes are pale grey with weak pigmentation, while the ears, muzzle and limbs are pale brownish, giving a subtly translucent appearance. Cranially, the skull is short (Fig. 8), with narrow zygomatic arches and a constricted postorbital region. The palate is smooth and lacks strong ridging and the molars are less robust than those of *C*.* sphinx*. Of four adult females captured in mid-elevation forests, two were lactating.

#### Family Rhinolophidae

Eight species of Rhinolophidae were documented. Four species (*R*.* episcopus*, *R*.* siamensis*, *R*. cf.* episcopus* and *R*.* perniger*) are newly recorded, while *R*.* affinis*, *R*.* thomasi*, *R*.* pearsonii* and *R*.* pusillus* had previously been reported by Nguyen et al. (2012). *R*.* rouxii*, listed in earlier surveys, was not detected. This species is now considered restricted to eastern Asia; therefore, its previous record from Xuan Nha NR is likely a misidentification. Reproductive evidence was observed in *R*.* thomasi* and *R*.* pearsonii*.

***Rhinolophus affinis*** **Horsfield, 1823**

**Figure 9.**
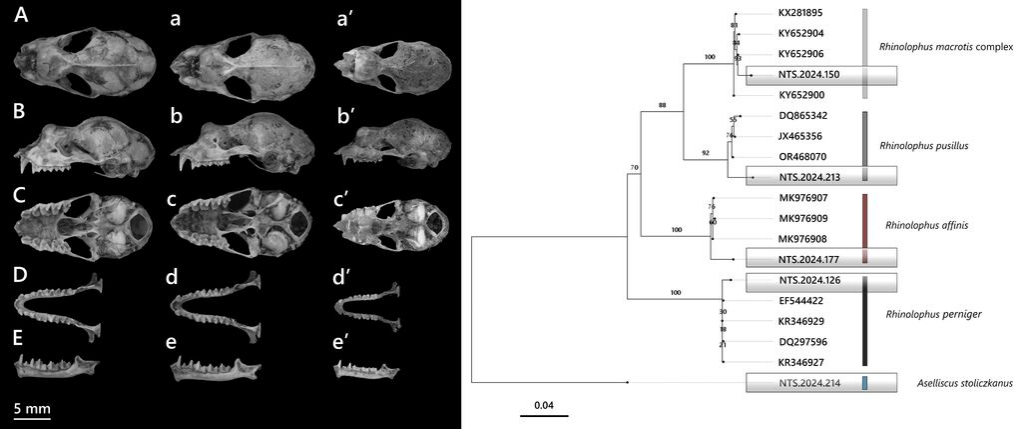
(**Left**) Dorsal (A, a, a’), lateral (B, b, b’), ventral (C, c, c’) views of the cranium and Dorsal (D, d, d’), lateral (E, e, e’) views of the mandible of *R*.* affinis*, *R*. *thomasi* and *R*. *pusillus*; (**Right**) ML tree based on Cyt *b* sequences of Rhinolophydae bats.

Only a female *R*.* affinis *was recorded showing no signs of reproductive activity. This species is a medium-sized horseshoe bat. Externally, it has a broad horseshoe with a deep median emargination, rounded connecting process and a moderately convex sella lacking basal lappets; the lancet is subtriangular with an unreduced tip. The pelage is soft, dark greyish-brown. The skull is small, with well-developed lateral nasal compartments and narrow interorbital constriction. The dentition shows a slightly reduced P^2^ within the tooth row. In comparison with *R*.* thomasi* (Fig. 9, left), although both species share skull shape typical of the “*R*.* megaphyllus*” group (Ith et al. 2015), *R*.* affinis* has a slightly larger cranium, a proportionally longer mandible, broader rostrum and more laterally expanded arches. Their dental dimensions (C^1^C^1^W, M^3^M^3^W) are otherwise similar. Molecular data place the specimen within the *R*.* affinis* clade, with pairwise genetic distances of 2.5–2.75% (Fig. 9, right).

***Rhinolophus thomasi*** **K. Andersen, 1905**

**Figure 10.**
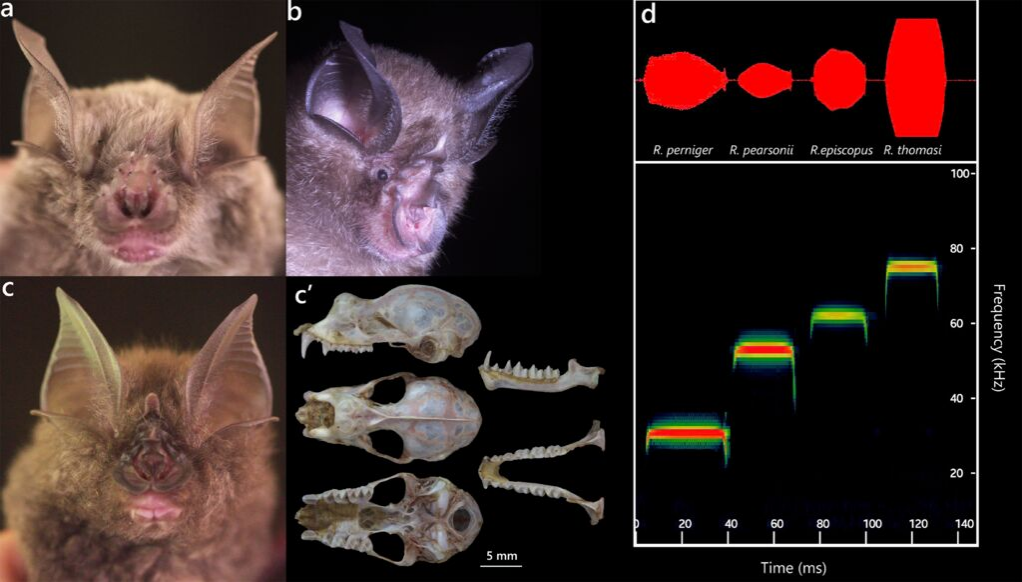
Selected Rhinolophydae species recorded: **(a) ***R*.* thomasi*; **(b) ***R*.* pusillus*; **(c) ***R*.* pearsonii*; **(c’) **Five aspects of the cranium and mandible of *R*.* pearsonii*; **(d) **Ultrasonic echolocation calls of Rhinolophids.

One lactating female of *R*.* thomasi* was observed. Compared to *R*.* affinis*, *R*.* thomasi* has slightly smaller zygomatic, braincase breadths and its mandible is thinner and shorter. Externally, it shows a narrow, rectangular horseshoe and a broad, bluntly pointed lancet. The pelage is uniformly light grey with a metallic sheen, dense and velvety, with pale bases and darker tips (Fig. 10a). Echolocation calls of a female exhibited broadband FM–CF–FM structure dominated by CF components, with a peak frequency of 77.4 ± 0.2 kHz, starting at 78.6 kHz and ending at 66.7 kHz. Calls averaged 19.6 ± 0.3 ms in duration, with the longest call lasting 20.3 ms (Fig. 10d).

***Rhinolophus pearsonii *Horsfield, 1851**

A total of 32 individuals of *R*.* pearsonii* were captured and were the most common species of *Rhinolophus* in the survey. Six females were reproductively active (five lactating, one pregnant). This species has a broad rostrum and a well-developed braincase. Cranial dimensions include GTL 22.78–23.85 mm, CM^3^L 8.97–9.78 mm and ML 16.27 ± 0.28 mm. The molars are marked by high, sharp cusps (Fig. 10c’). Echolocation calls of a male recorded in a tent exhibited broadband FM-CF-FM structure (Fig. 10d), with peak frequency 56.9 ± 0.7 kHz, ranging from 39.6 to 58.9 kHz. Calls averaged 21.6 ± 0.3 ms in duration, with the longest call lasting 22.3 ms.

***Rhinolophus perniger*** **Hodgson, 1843**

Previously, *R*.* perniger* was considered a subspecies within the *R*.* luctus* complex; however, studies of Volleth et al. (2017) demonstrated differences between *R*.* perniger* and *R*.* luctus* sensu stricto. In this study, a single female was captured and clustered with reference *R*.* perniger* sequences, showing 1.2% genetic divergence (Fig. 9, right). The individual was non-reproductive. *R*.* perniger* is a large horseshoe bat (FA 73.4 mm, Wt 48 g) with a massive skull (GTL 33.54 mm). The horseshoe-shaped nose-leaf is broad, covering the upper lip with a deep median notch. The fur is thick, dark grey dorsally and ventrally. Dentition is robust, with well-developed toothrows. Echolocation calls recorded from the captured female were broadband FM–CF–FM (Fig. 10d), with peak frequency 31.9 ± 0.7 kHz, starting at 32.4 kHz and ending at 22.7 kHz. Calls averaged 33.4 ± 0.7 ms in duration, with the longest call lasting 35.3 ms.

***Rhinolophus pusillus*** **Temminck, 1834**

This species is amongst the smallest horseshoe bats (Wt 4.2 g, FA 35.4 mm, CCL 11.54 mm), with proportionally small ears and nose-leaf and weakly developed supplementary leaflets (Fig. 10b). The lancet is elongated with a slight forward bend at the tip. The pelage is fine and silky, ranging from light brown to greyish-brown dorsally with a paler underside. The skull is small and delicate (Fig. 9, left), with a short, narrow rostrum, inflated braincase and slender zygomatic arches. The dentition is compact, with small, closely-spaced canines and premolars; the mandible is gently curved with diminutive lower teeth. In the phylogenetic analysis (Fig. 9, right), the specimen grouped within the *R*.* pusillus* clade; however, pairwise divergence from Vietnamese *R*.* pusillus* populations is high, ranging from 2.0% to 2.5%. This divergence shows a certain genetic differentiation of the studied individual relative to Vietnamese *R*.* pusillus*’s populations.

***Rhinolophus episcopus*** **Allen, 1923**

**Figure 11.**
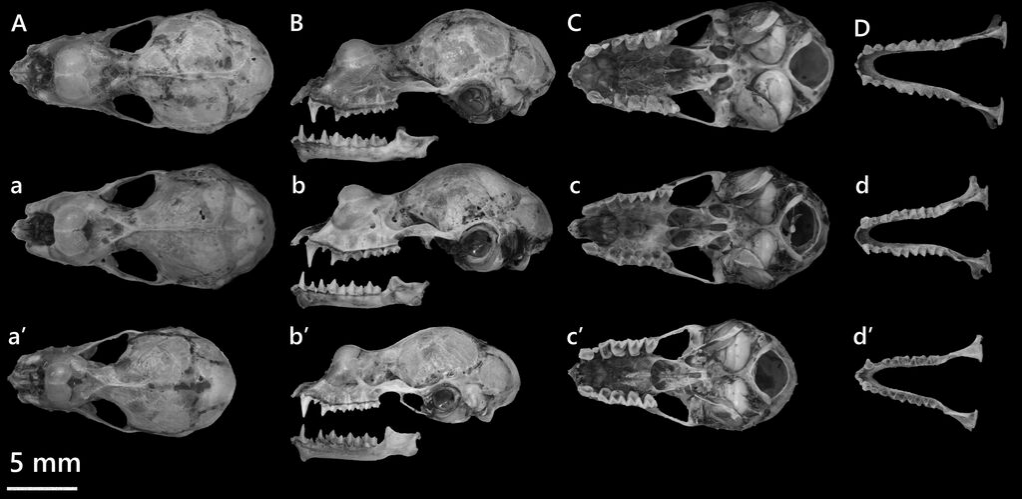
Dorsal (A, a, a’), lateral (B, b, b’), ventral (C, c, c’) views of the cranium and Dorsal (D, d, d’) views of the mandible of *R*.* episcopus*, *R*. cf. *episcopus* and *R*. *siamensis*, respectively.

The male *R*.* episcopus* has morphometric measurements: HB 41.8 mm, TL 21.9 mm, HF 8.5 mm, EL 23.4 mm, FA 42.1 mm and Wt 6 g. The bat has light brown pelage, large ears and a well-developed nose-leaf. The horseshoe is broad, covering the muzzle, with small lateral leaflets and a visible median notch. The lancet is elongated with convex margins and a rounded tip (Fig. 11). The anterior median swellings are prominent and elongated, while the posterior swellings are short. The sagittal crest is weakly developed and the frontal depression is shallow. Supraorbital crests are well-defined with sharp ridges.

In the *R*. *macrotis *complex, craniodental differences can be detected even amongst sympatric taxa. Comparative measurements between *R*.* episcopus* and *R*. cf.* episcopus* specimens collected from the same locality revealed that *R*.* episcopus* exhibits smaller CCL (13.79 mm vs. 14.16 mm) and BCH (6.73 mm vs. 6.95 mm) relative to *R*. cf. *episcopus*. Dental measurements such as CM^3^L (6.38 mm vs. 6.71 mm) and CP^4^L (2.85 mm vs. 3.05 mm) are smaller in *R*.* episcopus*. Conversely, *R*.* episcopus* displays a broader interorbital width (IOW). In this study, Cyt *b* sequencing was attempted for the specimen, but the obtained fragment (~ 500 bp) was insufficient in length and quality to be included in phylogenetic analyses. However, based on external morphology and craniodental traits, the identification of the specimen as *R*.* episcopus* is considered reliable. The echolocation call of this male was a broadband FM-CF-FM structure (Fig. 10d). The maximum start frequency was 65.6 kHz and the minimum value was 58.2 kHz, with peak energy at 63.4 ± 0.5 kHz. The mean call duration was 25.2 ± 0.6 ms, with the longest call lasting 26.8 ms.

***Rhinolophus siamensis*** **Gyldenstolpe, 1917**

*R*.* siamensis* initially described as a subspecies of *R*.* macrotis*, has since been elevated to species level, with a wide distribution in Southeast Asia (Vuong et al. 2017b, Hutson et al. 2019). This individual represents a small-sized member of the *macrotis* complex. The FA is 37.3 mm, HB is 40.7 mm and the Wt is only 3.07 g, all lower than corresponding values for *R*. cf.* episcopus*. The skull of *R*.* siamensis* is not only shorter, but also more gracile, shows a narrower braincase, reduced rostrum width and a shorter mandible (Table 3). Morphologically, *R*.* siamensis* has relatively large ears, about half the forearm length and a broad horseshoe covering the muzzle with a distinct median notch. The sella projects forwards and the connecting process is broad and rounded, giving the nose-leaf a structure distinct from other small horseshoe bats. Its pelage is soft and woolly, brown dorsally and paler ventrally.

***Rhinolophus* cf. *episcopus* Allen, 1923**

In this study, *R*. cf.* episcopus* was represented by a single adult male. External measurements included FA of 43.2 mm, E of 23.1 mm and Wt of 4.51 g. The ears were large, approximately half the length of the forearm and the pelage was soft, woolly and brown dorsally with a slightly paler ventral surface. The cranial profile is broad, with MAW of 8.91 mm and ZYW of 8.17 mm. The mandible is well-developed, with a ML of 10.12 mm. The skull morphology shows a more heavily built cranial structure compared to other members of the *macrotis* group (Fig. 11). Molecular analysis placed the specimen within the *R*.* macrotis* complex clade. It clustered most closely with individuals provisionally identified as *R*. cf.* episcopus* from Vietnam in the study of Vuong et al. 2017b, with a genetic divergence of approximately 2.5–3%. This level of divergence offers a close relationship, while also showing the possibility of regional differentiation within *R*. cf. episcopus (Liu et al. 2019).

#### Family Vespertilionidae

Several species reported by Nguyen et al. (2012), including *P*.* abramus*, *P*.* coromandra*, *P*.* javanicus*, *Ia io*, *T*.* fulvida *(referred to as *T*. *pachypus*) and *Murina cyclotis*, were not detected in the present survey. Nevertheless, our study expands the species inventory of Xuan Nha NR by providing new records for *T*.* tonkinensis* and *M*.* muricola*.

***Kerivoula * cf. *dongduongana * Vuong** **, Hassanin, Furey, Nguyen & Csorba, 2018**

According to Vuong et al. (2018) and other studies, *K*. *hardwickii* sensu lato in Vietnam has been divided into four smaller, closely-related species that are morphologically difficult to distinguish. Based on external and craniodental measurements, our specimen is tentatively assigned to *K*.* dongduongana*; however, molecular analysis is necessary to confirm its precise species identity. Morphologically, it shows soft, long fur, with smoky-brown dorsally and lighter greyish-brown ventrally. HB ranges 34.3–40.4 mm, FA 33.5–35.4 mm, with brownish-black translucent wing membranes. *K*. cf. *dongduongana* presents a domed braincase with a concave frontal profile. Cranial measurements (GTL 14.03–14.14 mm, ML 6.65–6.75 mm) point out a small and compact cranial structure compared to larger *Kerivoula* species (Vuong et al. 2018, Liang et al. 2023).

***Myotis alticraniatus *Osgood, 1932**

**Figure 12.**
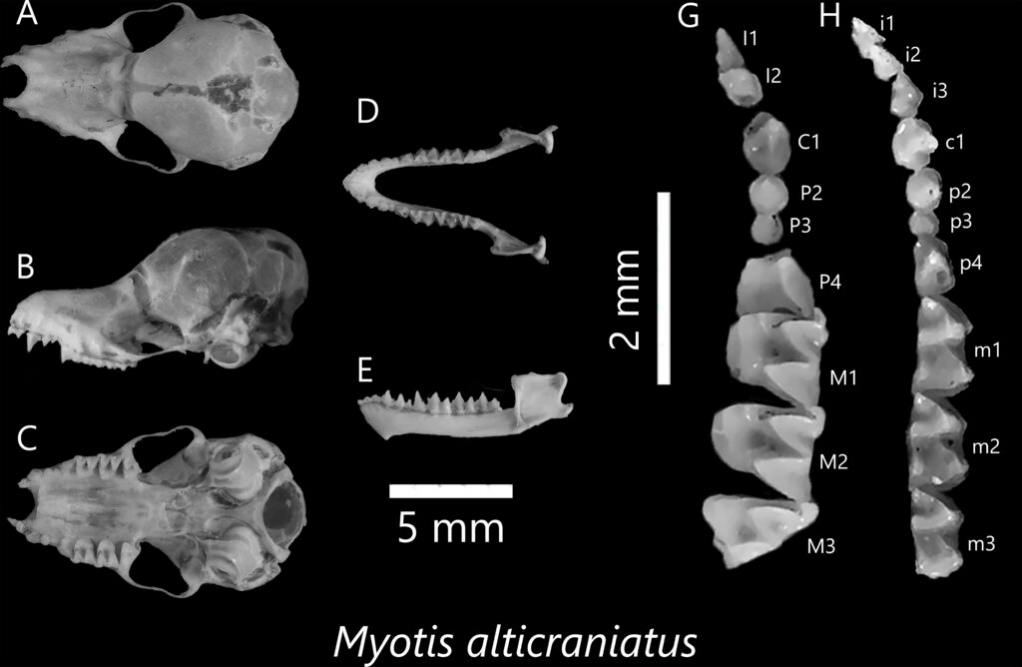
Dorsal (A), lateral (B), ventral view (C) of cranium; Dorsal (D), lateral (E) of mandibles, Occlusal view of left upper (G) and right lower (H) toothrows of *M*.* alticraniatus*.

*M*.* alticraniatus* was once considered a subspecies of *M*.* siligorensis* in Vietnam, but more recent taxonomic studies by Ruedi et al. (2021) have clarified its status as a distinct species. According to these studies, *M*.* siligorensis* is restricted to the Central and Eastern Himalayas, whereas bats occurring further east, including populations in China and Indochina, are now assigned to *M*.* alticraniatus*. A female specimen represents a well-preserved example of this small vespertilionid. It has a delicate skull with a low rostrum, domed braincase, steep frontal profile and slightly swollen occipital region. The zygomatic arches are thin and inwardly concave. Dorsal fur is buff to dark brown, with lighter greyish-brown ventrally. Ears are long and narrow, reaching or surpassing the muzzle tip. The upper premolars have a prominent size difference, particularly between P^2^ and P^4^ (Fig. 12). The bat has a delicate mandible with small lower premolars (p_4_) and its lower molars are semi-nyctalodont types.

***Myotis muricola *(Gray, 1864)**

**Figure 13.**
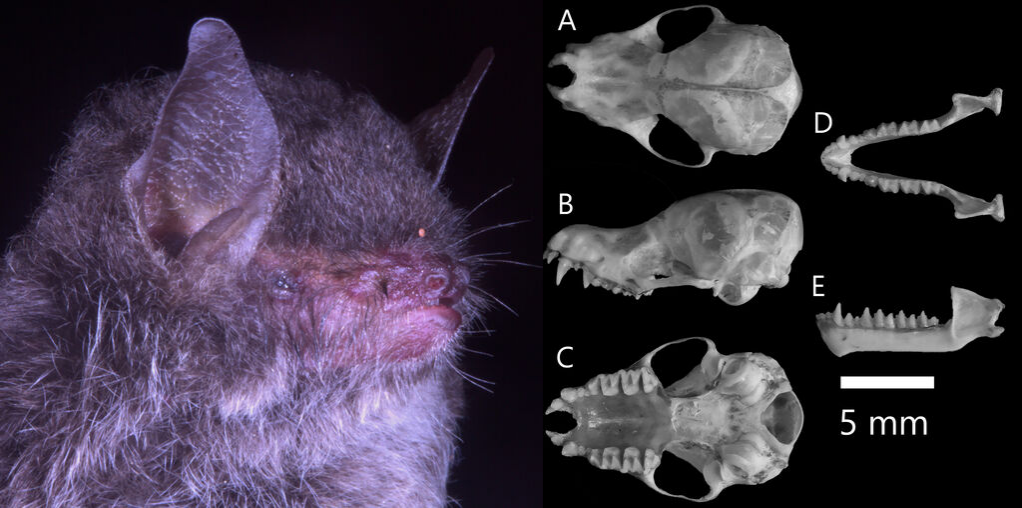
(**Left**) *M*.* muricola* recorded on Xuan Nha NR; (**Right**) Dorsal (A), lateral (B), ventral view (C) of cranium; Dorsal (D), lateral view (E) of mandibles, Occlusal view of left upper (G) and right lower (H) toothrows of *M*. *muricola*.

**Figure 14.**
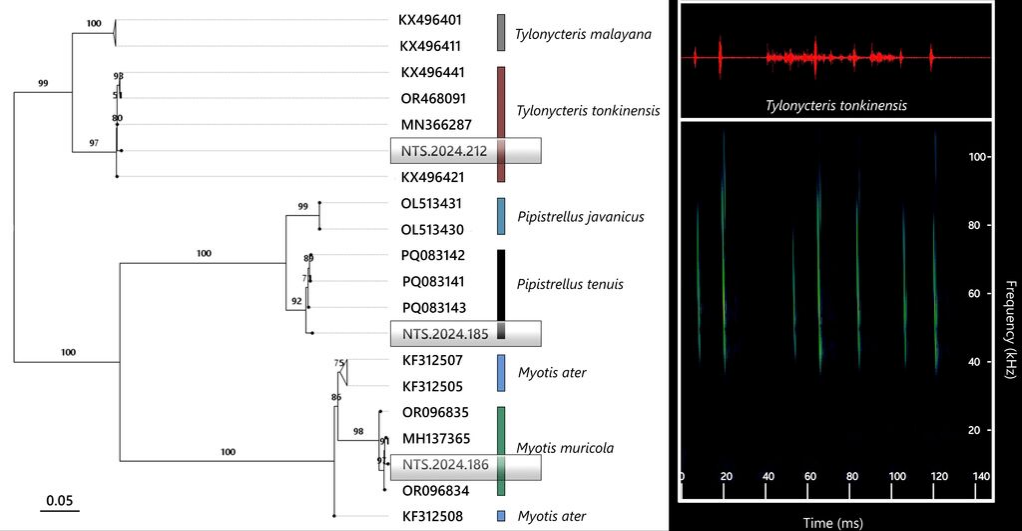
(**Left**) ML phylogenetic tree, based on Cyt *b *sequences of the recorded Vespertilionidae bats; nodes with BS < 70% are not displayed; (**Right**) Ultrasonic echolocation call characteristics of *T*.* tonkinensis*.

A female was collected at 950 m a.s.l. The pelage is soft and dense, pale brownish-grey dorsally and dirty white ventrally with dark hair roots (Fig. 13, left). Ears are long and narrow, the tragus slender and forward-bent and the feet small with short claws. The skull is small with an elongated rostrum, flattened braincase and developed sagittal crest (Fig. 13, right). The zygomatic arch is evenly curved and massive. C^1^ are wide and robust, exceeding the height of P^4^. P^2^ are well-developed and aligned in the tooth row. P^3^ are small, displaced lingually and stand inwards on the inside of the upper tooth row. c_1_ are large, pointed and slightly higher than p_4_. Lower molars are of the myotodont type. Genetic analysis of Cyt b confirmed the specimen within the *M*.* muricola* clade, with 1–1.5% divergence from other conspecific sequences (Fig. 14, left).

***Pipistrellus tenuis*** **(Temminck, 1840)**

**Figure 15.**
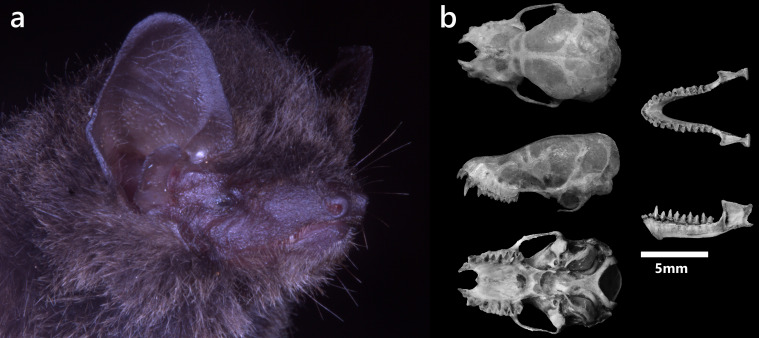
**(a) ***P*.* tenuis *recorded on Xuan Nha NR; **(b)** Five aspects of the cranium and mandible of *P*. *tenuis*.

Externally, *P*.* tenuis* has dark brown dorsal pelage with a slightly paler ventral side and a short broad tragus (Fig. 15a). The skull is small and delicate, with a narrow rostrum, inflated braincase, weak crests and simple bicuspid upper canines (Fig. 15b). Sequencing of the Cyt *b* gene positioned the specimen firmly within the *tenuis* clade. Pairwise genetic distances ranged from 1.0% to 1.7% compared to *P*.* tenuis* individuals previously collected from the Tay Con Linh Mountain by Kruskop et al. (2024) (Fig. 14, left).

***Tylonycteris tonkinensis*** **Tu, Csorba, Ruedi & Hassanin, 2017**

**Figure 16.**
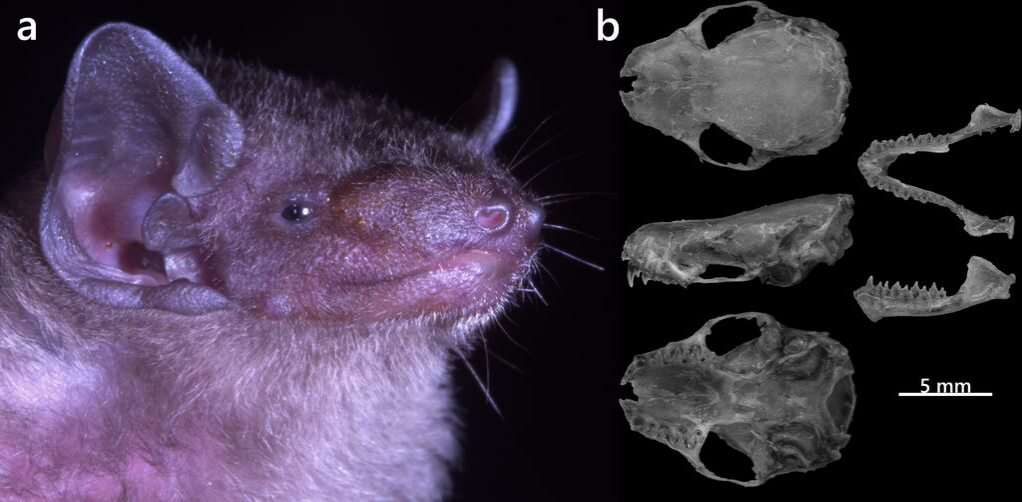
**(a) ***T*.* tonkinensis *recorded on Xuan Nha NR; **(b)** Five aspects of the cranium and mandible of *T*. *tonkinensis*.

Previously, Vietnamese populations were identified as *T*.* robustula*. However, integrative analyses combining genetic and morphological data by Vuong et al. (2017a) confirmed that these bats represent a distinct species, separated from the *robustula* lineage. Consequently, the taxon was formally described as *T*.* tonkinensis*. This very small bat (HB 45.2 mm, Wt 5.1 g) is distinguished by fleshy adhesive pads on the thumbs and soles, a short narrow tragus and uniform mid-brown pelage (Fig. 16a). The skull is flattened, with a depressed braincase and prominent supraorbital tubercles (Fig. 16b). The dentition displays distinctive features: I^2^ are twice smaller in height and crown area than I^1^, both bearing small supplementary cusps. The upper canine shows an additional cusp on its posterior blade. Lower molars have the talonid slightly exceeding the trigonid in M^1^ and M^2^.

Acoustic recordings revealed FM calls with peak energy at 58.2 ± 1.3 kHz, starting at 88.5 kHz and ending at 38.6 kHz (Fig. 14, right). The mean call duration was 2.1 ± 0.3 ms, with the longest call lasting 10.2 ms. Genetic analysis placed the specimen firmly within the *T*.* tonkinensis* clade, with only 0.35% divergence from published Vietnamese sequences (Fig. 14, left).

## Discussion

Our survey revealed a diverse bat assemblage, documented 19 bat species, expanding the known bat diversity in this ecosystem. The observed bat community structure is characterised by high species richness, but low evenness. This was due to the dominance of a few species, especially *H. poutensis* and *R. pearsonii*, which accounted for a disproportionate share of the captures. This skewed abundance distribution aligns with previous research on tropical bat populations, in which factors, such as habitat specialisation, roosting ecology and behavioural traits, strongly influence local species dominance (Furey et al. 2010, Kruskop 2013). Combined with the brief survey in 2010, the 2024 fieldwork shows improvements in sampling effort and increases in record diversity, emphasising the value of continuing to assess biodiversity in this region by integrating multiple approaches.

### Sampling constraints and implications for species detection

Although our findings indicate higher species richness compared to the previous survey by Nguyen et al. (2012), several limitations of our sampling design must be acknowledged, which may limit the completeness of our biodiversity assessment. Firstly, the lack of species accumulation curve or detection curve, primarily due to uneven capture rates and the limited trapping durations per site (only 1–2 nights), prevented more comprehensive richness estimations . Out of 19 species recorded, 11 were represented by only one individual, while a few species had sample sizes greater than three individuals. These low representation may reflect habitat specialisation, edge-of-range populations or insufficient sampling. This under-representation of most of these species limited the application of statistical methods to assess sampling completeness and potentially resulted in underestimation of actual species richness. This issue is consistent with patterns observed in other studies in Vietnam (Furey et al. 2010), which also noted that short trapping periods and low sample sizes preclude accurate assessment of bat diversity. The limited number of trapping nights and relatively short survey day, compared to more intensive, year-round or multi-seasonal studies, underscore the need for more extensive, longer-duration surveys to capture a complete overview of the local bat community. Additionally, early rainy season conditions during the survey period may have influenced bat activity patterns, resulting in lower detections compared to the peak rainy seasons, when insect prey availability and bat breeding activities typically peak (Furey et al. 2011, Kohles et al. 2024).

### Habitat specialisation, landscape modifications and distribution

Bat assemblages in Xuan Nha NR reflect complex interactions between ecological specialisation and landscape dynamics. High-elevation habitats with cooler microclimates support montane-adapted species (Kruskop and Shchinov 2010), while bamboo groves between 700 and 900 m a.s.l. may provide roosts for foliage-roosting vesper bats. Yet, anthropogenic disturbance through agricultural encroachment threatens these habitat-sensitive taxa and may promote the spread of generalist species (Abramov et al. 2009). Amongst these, *R*.* perniger *was found, a large-bodied species associated with mature forests, known to be highly sensitive to habitat degradation (Francis 2019). The consistent association of rare and habitat-specialist species, such as *H*.* griffini*, *R*.* perniger* and *R*. cf.* episcopus* emphasises the importance of conserving these habitats. Given the morphological conservatism of *R*. cf.* episcopus* and possible hidden diversity, further intergrative studies are needed to clarify species boundaries and detect cryptic taxa.

This study also provides the first distributional record of *H*.* griffini* in north-western Vietnam. Due to the common elevational range, it is likely that other individuals of this species may occur in adjacent regions, such as Xuan Lien and Ta Xua NR. Our survey also suggests that *H*.* griffini* and *H*.* armiger *are sympatric species due to our observations that they inhabit the same open forest and understorey habitat. However, *H*.* armiger* consistently dominates population size within the shared distribution range, which may indicate competitive advantages in roost selection.

### Integrative approaches to species identification

Our study employed the integrative approaches, combining external morphology, craniodental characters, preliminary genetic data and echolocation analysis to improve species identification. In this study, analyses, based on external morphology and craniodental characters, resulted in the classification of four morphological groups represented by four families. In particular, dentition characteristics, coronoid process structure and braincase height have been extensively demonstrated to be species-specific and can be considered as diagnostic characters, not only in bats (Borissenko and Kruskop 2003, Vuong et al. 2017b, Vu et al. 2024), but also in other small mammals, such as rodents and insectivores (Wilson and Mittermeier 2019, Bui et al. 2020b, Esquivel et al. 2021). However, some overlapping measurements amongst species for each group can lead to misidentifications. This problem is quite common in order Chiroptera, where morphological conservatism and the presence of cryptic taxa have significant challenges to accurate species identification (Francis 2019, Ruedi et al. 2021, Kruskop et al. 2024). Consequently, the application of integrated molecular techniques is needed to resolve taxonomic uncertainties. In addition to the morphological and genetic data, application of the echolocation approach has improved our knowledge of bat species composition in Xuan Nha NR. Echolocation were recorded in several species, facilitating species identification when morphology was insufficient to clearly differentiate between closely-related groups.

## Conclusions

Our findings strongly advocate the need for expanded multi-seasonal bat surveys at Xuan Nha NR to better study dynamics in species composition, reproductive phenology and habitat use. Given the moderate to high species richness observed and the potential for cryptic species, the ecosystems of Xuan Nha NR warrant further conservation priority. Our survey recorded *Hipposideros griffini*, a species listed as Near Threatened (NT) on the IUCN Red List due to ongoing habitat loss and its narrow distribution range. The presence of this species indicates the ecological significance of Xuan Nha NR and highlights the need for targeted management strategies to protect more taxa. Continued protection of mature forest patches and cave systems is critical to preserving the ecological integrity of this chiropteran community. Finally, our study contributes baseline data for the bat populations of north-western Vietnam and demonstrates the importance of integrating morphological, genetic and echolocation data in biodiversity assessments and conservation planning. We believe that continued research will significantly increase the known species richness of the region.

## Supplementary Material

074962C5-3DE6-5AA3-88EC-95EFDF4AAD91 10.3897/BDJ.13.e165516.suppl1 Supplementary material 1 Bat dataset table Data type occurences Brief description Bat dataset table from the field survey in Xuan Nha NR, Son La, Vietnam (March 2010 & August 2024). Dash (–): Not determined. Habitat nature: 1 = Evergreen forest, 2 = Disturbed secondary forest, 3 = Cave areas, 4 = Stream valley. File: oo_1429504.docx https://binary.pensoft.net/file/1429504 YHV, STN, HTB

E0C24727-3D73-50FA-AED7-FCCF4C1CF702 10.3897/BDJ.13.e165516.suppl2 Supplementary material 2 List of craniodental measurements used in this study Data type morphological File: oo_1355021.docx https://binary.pensoft.net/file/1355021 YHV, STN, HTB

## Figures and Tables

**Table 1.** List of bat species recorded from Xuan Nha NR. n (♂, ♀) = sample size (♂ = male, ♀ = female); Elevation: n (m) = number of individuals with elevation in metres. Recorded habitat: 1 = evergreen forest, 2 = disturbed secondary forest, 3 = cave areas, 4 = stream valley. Species records: ✓ = recorded, ✓* = newly recorded in the 2024, — = Not recorded.

**No.**	**Specific name**	**n (M♂, F♀)**	**Reproductive information**	**Elevation (m)**	**Habitat nature**	**Species recorded in this study**	**Previous Record (Nguyen et al. 2012)**	**IUCN Status**
**Hipposideridae Lydekker, 1891**								
1	*Hipposideros armiger* (Hodgson, 1835)	3 (2♂, 1♀)	1♀ (Not reproductive)	2 (650 m), 1 (750 m)	2	✓	✓	LC
2	*Hipposideros griffini* Vu, Puechmaille, Denzinger, Dietz, Csorba, Bates, Teeling & Schnitzler, 2012	1 (1♂, 0♀)	—	1 (650 m)	2	✓*	—	NT
3	*Hipposideros poutensis* Allen, 1906	39 (21♂, 18♀)	3♀ (3 Lactating), 15♀ (Not reproductive)	1 (600 m), 4 (650 m), 8 (700 m), 15 (750 m), 7 (800 m), 2 (850 m), 2 (910 m)	1, 2, 3, 4	✓	✓	LC
4	*Hipposideros gentilis* Andersen, 1918					—	✓	LC
5	*Aselliscus stoliczkanus* (Dobson, 1871)	3 (0♂, 3♀)	3♀ (Not reproductive)	1 (750 m), 2 (800 m)	1, 2	✓	✓	LC
**Pteropodidae Brisson, 1762**								
6	*Cynopterus sphinx* (Vahl, 1797)	8 (2♂, 6♀)	3♀ (2 Lactating, 1 Pregnant, 1 with newborn), 3♀ (Not reproductive)	1 (600 m), 5 (700 m), 2 (750 m)	2, 4	✓	✓	LC
7	*Megaerops niphanae* Yenbutra & Felten, 1983	7 (3♂, 4♀)	2♀ (2 Lactating), 2♀ (Not reproductive)	3 (600 m), 2 (700 m), 2 (750 m)	2, 4	✓*	—	LC
8	*Sphaerias blanfordi* (Thomas, 1891)					—	✓	LC
**Rhinolophidae Gray, 1825**								
9	*Rhinolophus affinis* Horsfield, 1823	1 (0♂, 1♀)	1♀ (Not reproductive)	1 (750 m)	2	✓	✓	LC
10	*Rhinolophus episcopus* Allen, 1923	1 (1♂, 0♀)	—	1 (600 m)	2	✓*	—	LC
11	*Rhinolophus siamensis* Gyldenstolpe, 1917	1 (1♂, 0♀)	—	1 (750 m)	2	✓*	—	LC
12	*Rhinolophus pearsonii* Horsfield, 1851	32 (5♂, 27♀)	6♀ (5 Lactating, 1 Pregnant), 21♀ (Not reproductive)	1 (500 m), 4 (600 m), 1 (650 m), 1 (700 m), 20 (750 m), 5 (800-850 m)	2, 4	✓	✓	LC
13	*Rhinolophus pusillus* Temminck, 1834	1 (1♂, 0♀)	—	1 (650 m)	2	✓	✓	LC
14	*Rhinolophus* cf. *episcopus* Allen, 1923	1 (1♂, 0♀)	—	1 (750 m)	2	✓*	—	LC
15	*Rhinolophus thomasi* K. Andersen, 1905	7 (5♂, 2♀)	1♀ (1 Lactating), 1♀ (Not reproductive)	2 (600 m), 5 (800 m)	2, 4	✓	✓	LC
16	*Rhinolophus rouxii* Temminck, 1835					—	✓	LC
17	*Rhinolophus perniger* Hodgson, 1843	1 (0♂, 1♀)	1♀ (Not reproductive)	1 (600 m)	2	✓*	—	LC
**Vespertilionidae Gray, 1821**								
18	*Kerivoula *cf. dongduongana Vuong et al. 2018	4 (0♂, 4♀)	4♀ (Not reproductive)	4 (900 m)	1	✓	✓	LC
19	*Murina cyclotis* Dobson, 1872					—	✓	LC
20	*Myotis alticraniatus* Osgood, 1932	1 (0♂, 1♀)	1♀ (Not reproductive)	1 (800 m)	2	✓	✓	LC
21	*Myotis muricola* (Gray, 1864)	1 (0♂, 1♀)	1♀ (Not reproductive)	1 (950 m)	2	✓*	—	LC
22	*Pipistrellus tenuis* (Temminck, 1840)	1 (1♂, 0♀)	—	1 (800 m)	2	✓	✓	LC
23	*Pipistrellus abramus* (Temminck, 1840)					—	✓	LC
24	*Pipistrellus coromandra* Gray, 1838					—	✓	LC
25	*Pipistrellus javanicus* Gray, 1838					—	✓	LC
26	*Ia io* Thomas, 1902					—	✓	NT
27	*Tylonycteris pachypus* (Temminck, 1840)					—	✓	LC
28	*Tylonycteris tonkinensis* Tu, Csorba, Ruedi & Hassanin, 2017	1 (1♂, 0♀)	—	1 (600 m)	4	✓*	—	LC

**Table 2.** External measurements of bat specimens. Values are presented as min–max (upper row) and mean ± SD (lower row, if n ≥ 3). All measurements are in millimetres, except Wt (grams).

**Character**	**HB**	**T**	**HF**	**E**	**FA**	**Wt**
*H*.* armiger*	87.7–95.7 92.16±3.07	62.6–68.2 64.71±3.84	17.7–17.8 17.73±0.06	30.7–32.7 31.73±1	92.6–98.9 94.73±5.42	47–66 57.67±6.28
*H*.* griffini*	88.8	60.3	16.3	28.3	87.6	54
*H*.* poutensis*	59.5–71.1 65.20±2.93	30.5–47.7 37.24±3.05	8.4–14 10.72±1.12	11.6–29.3 21.53±2.9	54.7–61 58.44±1.56	10.25–19.5 14.52±1.88
*A*.* stoliczkanus*	40.7–45.4 43.07±2.35	35.6–39.4 37.53±1.9	6.3–6.9 6.63±0.31	7.7–10.2 9.20±1.32	41.3–42.5 42.07±0.67	4.5–6.3 5.12±1.03
*C*.* sphinx*	79.2–97.8 89.94±6.68	11–14.4 12.21±1.33	11.6–16.4 14.43±1.55	18.9–23.3 21.00±1.46	68.2–73.5 71.15±1.89	37–61 45.63±8.81
*M*.* niphanae*	70–87.6 80.64±5.87	—	9.8–12.6 11.20±0.88	15.6–17.1 16.56±0.48	54.8–58.6 56.57±1.56	26.15–32 28.41±2.02
*R*.* affinis*	50.2	22.3	7.8	18.2	46.2	8.1
*R*.* episcopus*	41.8	21.9	8.5	23.4	42.1	6
*R*.* siamensis*	40.7	18.4	7.3	19.3	37.3	3.07
*R*.* pearsonii*	50.4–64.3 60.31±2.55	10.1–27.2 20.23±2.92	8.8–13.1 11.28±0.94	15.8–25.3 22.62±2.41	48.1–54.8 52.23±1.53	8.85–13 10.24±1.03
*R*.* pusillus*	39.8	23.9	7.2	15.6	35.4	4.2
*R*. cf. *episcopus*	44.7	18.1	8.2	23.1	43.2	4.51
*R*.* thomasi*	45.1–53.2 48.79±2.51	20.8–27.6 23.09±2.41	7.1–8.5 7.89±0.43	14.7–17.8 16.74±1.21	38–43.9 42.38±2.43	6.3–8.5 7.12±0.8
*R*.* perniger*	83.2	59.5	18.4	37.5	73.4	48
*K*. cf. *dongduongana*	34.3–40.4 37.90±2.64	39.8–41.6 40.88±0.76	6.5–7.5 6.95±0.42	12.9–14.5 13.33±0.78	33.5–35.4 34.55±0.79	4.6–5 4.83±0.21
*M*.* alticraniatus*	41.6	36.72	5.3	10.17	31.8	2.7
*M*.* muricola*	45.3	45.8	62	12.1	36.5	3.4
*P*.* tenuis*	41.1	31.5	5.6	10.9	28.1	2.6
*T*.* tonkinensis*	45.2	28.2	5.7	10.2	25.7	5.1

**Table 3.** Craniodental measurements with abbreviations shown in Suppl. material 2. Min–max values in upper row and mean, standard deviation (if n ≥ 3) in lower row are given. The dash (–) indicates characters not taken in this study.

**Character**	**Species** **(n)**						
	***H*.* armiger*** **(3)**	***H*.** *** griffini*** **(1)**	***H*.** *** poutensis*** **(39)**	***A*.** *** stoliczkanus*** **(3)**	***C*.** *** sphinx*** **(8)**	***M*.** *** niphanae*** **(7)**	
**GTL**	31.15–32.79 32.10±0.85	30.11	22.34–23.26 22.86±0.24	14.81–15.19 15.04±0.2	30.47–33.09 31.47±0.84	26.93–27.75 27.31±0.27	
**CCL**	28.87–31.26 30.07±1.27	26.78	17.21–18.21 17.77±0.21	10.81–11.21 10.97±0.21	25.08–27.11 25.91±0.71	21.77–23.68 22.66±0.61	
**CM^3^L**	12.45–13.05 12.78±0.31	11.42	8.45–9.03 8.71±0.13	4.96–5.19 5.09±0.12	10.32–11.17 10.58±0.28	7.98–8.62 8.36±0.26	
**CP^4^L**	5.48–5.61 5.55±0.11	5.31	3.63–4.17 3.90±0.15	2.03–2.17 2.11±0.07	7.77–8.67 8.07±0.28	6.05–6.4 36.28±0.14	
**P^4^M^3^L**	9.38–9.45 9.42±0.04	8.98	5.96–6.57 6.32±0.13	3.63–3.88 3.74±0.13	4.34–4.78 4.59±0.15	3.56–4.17 3.84±0.23	
**M^1^M^3^L**	7.54–7.81 7.71±0.15	7.39	4.71–5.35 5.07±0.14	2.98–3.09 3.05±0.06	2.11–2.47 2.22±0.11	1.65–2.11 1.80±0.16	
**MAW**	15.24–15.94 15.58±0.35	15.45	10.71–11.69 11.27±0.21	7.03–7.33 7.16±0.16	—	—	
**BCH**	11.65–12.13 11.85±0.25	11.45	6.86–7.53 7.18±0.16	5.05–5.13 5.08±0.04	9.96–11.99 10.74±0.64	9.85–10.61 10.16±0.24	
**BB**	13.63–14.26 13.88±0.34	13.67	9.56–10.31 10.01±0.22	6.11–6.55 6.26±0.25	14.13–15.21 14.62±0.36	12.58–13.53 13.11±0.31	
**RW**	9.45–9.75 9.58±0.15	9.14	6.44–7.0 96.76±0.15	4.03–4.47 4.25±0.22	—	—	
**IOW**	4.32–4.83 4.55±0.26	3.78	3.18–3.57 3.35±0.12	1.69–1.83 1.77±0.07	6.36–7.27 6.63±0.28	5.78–6.85 6.25±0.38	
**ZYW**	18.02–18.21 18.13±0.1	17.62	12.32–13.11 12.74±0.19	7.31–7.61 7.43±0.16	18.88–21.11 19.74±0.67	17.37–18.31 17.91±0.34	
**C^1^C^1^W**	8.53–8.72 8.63±0.1	8.11	5.01–5.78 5.51±0.17	3.19–3.27 3.22±0.04	6.32–7.65 6.76±0.44	5.17–5.84 5.44±0.25	
**M^3^M^3^W**	12.84–12.95 12.88±0.06	11.65	8.28–9.04 8.63±0.16	5.01–5.18 5.10±0.09	8.98–10.05 9.59±0.38	7.91–8.52 8.19±0.22	
**ML**	21.71–23.22 22.54±0.77	20.23	14.79–15.81 15.34±0.21	9.21–9.33 9.27±0.06	23.39–24.57 23.79±0.42	19.24–20.25 19.78±0.34	
**CPH**	6.51–7.71 7.09±0.6	6.31	4.01–4.78 4.34±0.14	2.02–2.08 2.05±0.03	11.27–13.17 12.31±0.71	10.08–11.12 10.60±0.37	
**cm_3_L**	13.48–14.21 13.94±0.4	12.69	8.98–9.61 9.32±0.15	5.31–5.38 5.34±0.04	11.37–12.35 11.67±0.33	8.91–9.58 9.30±0.25	
**cp_4_L**	4.94–5.17 5.08±0.12	4.71	3.12–3.59 3.35±0.11	1.71–1.72 1.71±0.01	7.35–8.24 7.69±0.31	5.72–6.2 55.98±0.2	
**p_4_m_3_L**	10.55–10.84 10.67±0.15	10.12	6.75–7.31 7.04±0.13	4.05–4.16 4.10±0.06	6.05–6.43 6.25±0.12	5.01–5.53 5.23±0.17	
**m_1_m_3_L**	8.72–8.95 8.87±0.13	8.36	5.53–6.07 5.82±0.14	3.45–3.54 3.49±0.05	3.64–3.98 3.82±0.12	2.95–3.47 3.20±0.17	
**Character**	**Species** **(n)**						
	***R*** **.** *** affinis*** **(1)**	***R*** **.** *** episcopus*** **(1)**	***R*** **.** *** siamensis*** **(1)**	***R*** **.** *** pearsonii*** **(32)**	***R*** **.** *** pusillus*** **(1)**	***R*** **.** **cf.*episcopus*** **(1)**	
**GTL**	18.96	17.69	15.66	22.78–23.85 23.29±0.3	15.01	17.76	
**CCL**	14.95	13.79	12.29	18.14–19.19 18.66±0.27	11.54	14.16	
**CM^3^L**	7.29	6.38	5.76	8.97–9.78 9.32±0.21	5.34	6.71	
**CP^4^L**	3.25	2.85	2.46	4.07–4.69 4.27±0.16	1.84	3.05	
**P^4^M^3^L**	5.31	4.57	4.21	6.32–7.08 6.68±0.16	3.95	4.88	
**M^1^M^3^L**	4.45	3.77	3.44	5.01–5.73 5.38±0.16	3.18	3.87	
**MAW**	9.18	8.97	7.83	10.48–11.12 10.80±0.19	7.33	8.91	
**BCH**	6.94	6.73	5.83	7.31–7.93 7.61±0.14	5.89	6.95	
**BB**	8.82	7.93	7.09	9.94–10.84 10.24±0.22	6.76	7.96	
**RW**	5.42	4.83	3.96	5.69–6.21 5.99±0.13	3.99	5.09	
**IOW**	2.79	2.51	2.07	2.11–2.84 2.43±0.16	2.17	2.41	
**ZYW**	9.97	7.97	7.29	11.08–12.09 11.61±0.25	7.27	8.17	
**C^1^C^1^W**	4.98	3.88	3.39	5.72–6.49 6.09±0.17	3.42	3.62	
**M^3^M^3^W**	7.34	5.83	5.01	8.33–9.18 8.71±0.23	5.33	5.71	
**ML**	12.77	11.29	9.05	15.89–16.89 16.27±0.28	9.73	10.12	
**CPH**	2.75	2.35	1.99	3.11–3.65 3.39±0.12	1.82	1.98	
**cm_3_L**	7.61	6.64	5.37	9.57–10.42 9.98±0.21	5.68	5.94	
**cp_4_L**	2.79	2.22	1.67	3.58–4.13 3.84±0.15	1.93	2.21	
**p_4_m_3_L**	5.66	4.85	4.13	7.01–7.74 7.37±0.18	4.28	4.43	
**m_1_m_3_L**	4.98	4.02	3.46	5.73–6.38 6.03±0.15	3.64	3.81	
**Character**	**Species** **(n)**						
	***R*.** *** thomasi*** **(7)**	***R*.** *** perniger*** **(1)**	***K*.** **cf. *dongduongana*** **(4)**	***M*.** *** alticraniatus*** **(1)**	***M*.** *** muricola*** **(1)**	***P*.** *** tenuis*** **(1)**	***T*.** *** tonkinensis*** **(1)**
**GTL**	18.58–19.01 18.83±0.16	33.54	14.03–14.14 14.09±0.06	12.35	14.21	12.34	12.65
**CCL**	14.61–14.91 14.76±0.15	26.23	12.42–12.58 12.52±0.07	9.34	12.21	9.74	10.13
**CM^3^L**	7.02–7.35 7.15±0.13	12.59	5.05–5.19 5.14±0.06	4.38	5.35	4.14	3.92
**CP^4^L**	3.21–3.31 3.24±0.03	5.72	2.55–2.58 2.56±0.01	2.15	2.45	1.88	1.56
**P^4^M^3^L**	5.29–5.49 5.35±0.07	8.98	3.37–3.54 3.45±0.08	3.08	4.03	3.22	3.06
**M^1^M^3^L**	4.21–4.55 4.38±0.13	7.32	—	2.61	3.22	2.71	2.53
**MAW**	9.06–9.24 9.17±0.07	—	7.38–7.58 7.45±0.09	6.27	7.13	6.53	7.03
**BCH**	6.88–7.29 7.04±0.17	10.24	4.42–4.54 4.50±0.05	4.71	4.86	4.35	3.17
**BB**	8.62–8.94 8.78±0.12	13.05	6.65–6.75 6.69±0.05	5.95	6.75	6.13	4.45
**RW**	5.16–5.49 5.32±0.13	8.43	—	—	—	4.07	—
**IOW**	2.39–2.79 2.61±0.17	3.14	3.24–3.52 3.36±0.12	3.01	3.22	3.24	3.85
**ZYW**	9.58–9.97 9.73±0.14	15.66	7.93–8.31 8.16±0.16	6.97	8.83	7.52	8.96
**C^1^C^1^W**	4.59–4.98 4.78±0.15	8.69	3.19–3.32 3.27±0.06	2.98	3.52	3.71	3.91
**M^3^M^3^W**	7.14–7.34 7.25±0.07	11.18	5.06–5.2 5.14±0.06	4.55	5.79	5.02	5.38
**ML**	12.57–12.79 12.68±0.08	22.98	9.13–9.49 9.31±0.16	8.68	10.43	8.54	8.81
**CPH**	2.61–2.73 2.66±0.05	5.72	2.83–3.05 2.95±0.1	2.06	3.11	2.24	2.42
**cm_3_L**	7.33–7.69 7.54±0.13	13.48	5.42–5.51 5.47±0.04	4.66	5.65	4.45	4.23
**cp_4_L**	2.32–2.79 2.55±0.17	5.38	2.33–2.39 2.36±0.03	1.87	2.07	1.43	1.21
**p_4_m_3_L**	5.67–6.07 5.86±0.16	10.03	—	3.27	4.02	3.41	3.12
**m_1_m_3_L**	4.93–5.18 5.05±0.1	8.16	—	2.95	3.37	2.85	2.81
