# Supplementary material for: Study of bat diversity (Mammalia, Chiroptera) in Xuan Nha Nature Reserve, Son La Province, north-western Vietnam, based on integrative insights from morphology, genetics and echolocation data
Source: Biodivers Data J. 2025 Nov 4;13:e165516. doi: 10.3897/BDJ.13.e165516 (PMC12606075; doi:10.3897/BDJ.13.e165516)
Supplement: Supplementary material 1 — Bat dataset table [file bdj-13-e165516-s001.docx]

**Supplementary material 1.** Individual-based bat dataset table from the field survey in Xuan Nha Nature Reserve, Son La, Vietnam (March 2010 & August 2024). Dash (–): Not determined at the study sites. Habitat nature: 1 = Evergreen forest, 2 = Disturbed secondary forest, 3 = Cave areas, 4 = Stream valley

| Habitat nature: 1 = Evergreen forest, 2 = Disturbed secondary forest, 3 = Cave areas, 4 = Stream valley | | | | | | | | | | |  |
| --- | --- | --- | --- | --- | --- | --- | --- | --- | --- | --- | --- |
| **No.** | **Time** | **Coordinate** | **Elevation** | **Habitat nature** | **Trap** | **Recorded echolocation** | **Voucher** | ***Species*** | **Sex** | **Reproductive status** |  |
|  |  |  |  |  |  |  |  |  |  |  |  |
| 1 | 5/8/2024 | 20°42′54″N, 104°41′38″E | 650 | 2 | Harp |  | NTS.2024.117 | *Aselliscus stoliczkanus* | F | non-reproductive |  |
| 2 | 5/8/2024 | 20°42'57"N, 104°41'34"E | 650 | 2 | Mist net | ✓ | NTS.2024.118 | *Hipposideros armiger* | M | unknown |  |
| 3 | 5/8/2024 | 20°42'57"N, 104°41'34"E | 650 | 2 | Mist net | ✓ | NTS.2024.121 | *Hipposideros armiger* | M | unknown |  |
| 4 | 5/8/2024 | 20°42′54″N, 104°41′38″E | 650 | 2 | Harp | ✓ | NTS.2024.115 | *Hipposideros poutensis* | F | non-reproductive |  |
| 5 | 5/8/2024 | 20°42′54″N, 104°41′38″E | 650 | 2 | Harp | ✓ | NTS.2024.116 | *Hipposideros poutensis* | M | unknown |  |
| 6 | 5/8/2024 | 20°41′26″N, 104°41′12″E | 600 | 2 | Harp | ✓ | NTS.2024.119 | *Rhinolophus episcopus* | M | unknown |  |
| 7 | 5/8/2024 | 20°41′26″N, 104°41′12″E | 600 | 2 | Harp |  | NTS.2024.122 | *Rhinolophus pearsonii* | F | non-reproductive |  |
| 8 | 5/8/2024 | 20°41′26″N, 104°41′12″E | 600 | 2 | Harp | ✓ | NTS.2024.120 | *Rhinolophus thomasi* | M | unknown |  |
| 9 | 6/8/2024 | 20°42'57"N, 104°41'34"E | 650 | 2 | Mist net | ✓ | NTS.2024.123 | *Hipposideros griffini* | M | mature |  |
| 10 | 6/8/2024 | 20°42′28″N, 104°41′42″E | 750 | 4 | Harp | ✓ | NTS.2024.128 | *Hipposideros poutensis* | M | unknown |  |
| 11 | 6/8/2024 | 20°43′15″N, 104°41′38″E | 600 | 2 | Harp |  | NTS.2024.124 | *Hipposideros poutensis* | M | unknown |  |
| 12 | 6/8/2024 | 20°42′54″N, 104°41′42″E | 750 | 4 | Mist net |  | NTS.2024.127 | *Megaerops niphanae* | M | unknown |  |
| 13 | 6/8/2024 | 20°43′15″N, 104°41′38″E | 600 | 2 | Harp | ✓ | NTS.2024.125 | *Rhinolophus thomasi* | F | non-reproductive |  |
| 14 | 6/8/2024 | 20°43′15″N, 104°41′38″E | 600 | 2 | Harp | ✓ | NTS.2024.126 | *Rhinolophus perniger* | F | non-reproductive |  |
| 15 | 7/8/2024 | 20°41′51″N, 104°41′35″E | 850 | 4 | Mist net |  | NTS.2024.132 | *Hipposideros poutensis* | F | non-reproductive |  |
| 16 | 7/8/2024 | 20°42′54″N, 104°41′17″E | 750 | 2 | Harp |  | NTS.2024.133 | *Hipposideros poutensis* | F | non-reproductive |  |
| 17 | 7/8/2024 | 20°42′54″N, 104°41′17″E | 750 | 2 | Harp |  | NTS.2024.134 | *Hipposideros poutensis* | F | lactating |  |
| 18 | 7/8/2024 | 20°42′54″N, 104°41′17″E | 750 | 2 | Harp |  | NTS.2024.144 | *Hipposideros poutensis* | F | non-reproductive |  |
| 19 | 7/8/2024 | 20°41′51″N, 104°41′35″E | 850 | 4 | Mist net |  | NTS.2024.131 | *Hipposideros poutensis* | M | unknown |  |
| 20 | 7/8/2024 | 20°42′28″N, 104°41′42″E | 750 | 4 | Harp |  | NTS.2024.129 | *Hipposideros poutensis* | M | unknown |  |
| 21 | 7/8/2024 | 20°42′28″N, 104°41′42″E | 750 | 4 | Harp |  | NTS.2024.130 | *Hipposideros poutensis* | M | unknown |  |
| 22 | 7/8/2024 | 20°42′27″N, 104°41′31″E | 750 | 2 | Harp |  | NTS.2024.141 | *Rhinolophus pearsonii* | F | non-reproductive |  |
| 23 | 7/8/2024 | 20°42′27″N, 104°41′31″E | 750 | 2 | Harp |  | NTS.2024.142 | *Rhinolophus pearsonii* | F | non-reproductive |  |
| 24 | 7/8/2024 | 20°42′27″N, 104°41′31″E | 750 | 2 | Harp |  | NTS.2024.143 | *Rhinolophus pearsonii* | F | non-reproductive |  |
| 25 | 7/8/2024 | 20°42′54″N, 104°41′17″E | 750 | 2 | Harp |  | NTS.2024.135 | *Rhinolophus pearsonii* | F | non-reproductive |  |
| 26 | 7/8/2024 | 20°42′54″N, 104°41′17″E | 750 | 2 | Harp |  | NTS.2024.145 | *Rhinolophus pearsonii* | F | lactating |  |
| 27 | 7/8/2024 | 20°42′54″N, 104°41′42″E | 750 | 4 | Mist net |  | NTS.2024.137 | *Rhinolophus pearsonii* | F | non-reproductive |  |
| 28 | 7/8/2024 | 20°42′54″N, 104°41′42″E | 750 | 4 | Mist net |  | NTS.2024.138 | *Rhinolophus pearsonii* | F | non-reproductive |  |
| 29 | 7/8/2024 | 20°43′15″N, 104°41′38″E | 600 | 2 | Harp |  | NTS.2024.136 | *Rhinolophus pearsonii* | M | unknown |  |
| 30 | 7/8/2024 | 20°43′15″N, 104°41′38″E | 600 | 2 | Harp |  | NTS.2024.139 | *Rhinolophus pearsonii* | F | non-reproductive |  |
| 31 | 7/8/2024 | 20°43′15″N, 104°41′38″E | 600 | 2 | Harp |  | NTS.2024.140 | *Rhinolophus pearsonii* | F | non-reproductive |  |
| 32 | 8/8/2024 | 20°42′54″N, 104°41′17″E | 750 | 2 | Harp |  | NTS.2024.147 | *Aselliscus stoliczkanus* | F | non-reproductive |  |
| 33 | 8/8/2024 | 20°40′28″N, 104°41′07″E | 750 | 2 | Mist net |  | NTS.2024.159 | *Cynopterus sphinx* | F | non-reproductive |  |
| 34 | 8/8/2024 | 20°42′54″N, 104°41′42″E | 750 | 4 | Mist net |  | NTS.2024.146 | *Megaerops niphanae* | F | lactating |  |
| 35 | 8/8/2024 | 20°42′54″N, 104°41′17″E | 750 | 2 | Harp |  | NTS.2024.150 | *Rhinolophus* cf. *episcopus* | M | unknown |  |
| 36 | 8/8/2024 | 20°39′58″N, 104°40′55″E | 800 | 2 | Harp |  | NTS.2024.156 | *Rhinolophus pearsonii* | F | lactating |  |
| 37 | 8/8/2024 | 20°39′58″N, 104°40′55″E | 800 | 2 | Harp |  | NTS.2024.163 | *Rhinolophus pearsonii* | F | non-reproductive |  |
| 38 | 8/8/2024 | 20°39′58″N, 104°40′55″E | 800 | 2 | Harp |  | NTS.2024.164 | *Rhinolophus pearsonii* | F | lactating |  |
| 39 | 8/8/2024 | 20°40′28″N, 104°41′07″E | 750 | 2 | Mist net |  | NTS.2024.157 | *Rhinolophus pearsonii* | F | non-reproductive |  |
| 40 | 8/8/2024 | 20°40′28″N, 104°41′07″E | 750 | 2 | Mist net |  | NTS.2024.161 | *Rhinolophus pearsonii* | M | unknown |  |
| 41 | 8/8/2024 | 20°40′28″N, 104°41′07″E | 750 | 2 | Mist net |  | NTS.2024.162 | *Rhinolophus pearsonii* | F | non-reproductive |  |
| 42 | 8/8/2024 | 20°42′54″N, 104°41′17″E | 750 | 2 | Harp |  | NTS.2024.148 | *Rhinolophus pearsonii* | F | non-reproductive |  |
| 43 | 8/8/2024 | 20°42′54″N, 104°41′17″E | 750 | 2 | Harp |  | NTS.2024.149 | *Rhinolophus pearsonii* | M | unknown |  |
| 44 | 8/8/2024 | 20°42′54″N, 104°41′17″E | 750 | 2 | Harp |  | NTS.2024.152 | *Rhinolophus pearsonii* | F | lactating |  |
| 45 | 8/8/2024 | 20°42′54″N, 104°41′17″E | 750 | 2 | Harp |  | NTS.2024.153 | *Rhinolophus pearsonii* | F | non-reproductive |  |
| 46 | 8/8/2024 | 20°42′54″N, 104°41′17″E | 750 | 2 | Harp |  | NTS.2024.154 | *Rhinolophus pearsonii* | F | pregnant |  |
| 47 | 8/8/2024 | 20°42′54″N, 104°41′17″E | 750 | 2 | Harp |  | NTS.2024.155 | *Rhinolophus pearsonii* | F | non-reproductive |  |
| 48 | 8/8/2024 | 20°42′54″N, 104°41′17″E | 750 | 2 | Harp |  | NTS.2024.151 | *Rhinolophus siamensis* | M | unknown |  |
| 49 | 8/8/2024 | 20°41′50″N, 104°40′59″E | 800 | 2 | Mist net |  | NTS.2024.158 | *Rhinolophus thomasi* | M | unknown |  |
| 50 | 8/8/2024 | 20°41′50″N, 104°40′59″E | 800 | 2 | Mist net |  | NTS.2024.160 | *Rhinolophus thomasi* | M | unknown |  |
| 51 | 9/8/2024 | 20°40′28″N, 104°41′07″E | 750 | 2 | Mist net |  | NTS.2024.194 | *Cynopterus sphinx* | F | lactating |  |
| 52 | 9/8/2024 | 20°40′28″N, 104°41′07″E | 750 | 2 | Mist net |  | NTS.2024.179 | *Hipposideros armiger* | F | non-reproductive |  |
| 53 | 9/8/2024 | 20°39′58″N, 104°40′55″E | 800 | 1 | Harp |  | NTS.2024.165 | *Hipposideros poutensis* | F | non-reproductive |  |
| 54 | 9/8/2024 | 20°39′58″N, 104°40′55″E | 800 | 1 | Harp |  | NTS.2024.166 | *Hipposideros poutensis* | F | non-reproductive |  |
| 55 | 9/8/2024 | 20°41′50″N, 104°40′59″E | 800 | 1 | Harp |  | NTS.2024.167 | *Hipposideros poutensis* | F | non-reproductive |  |
| 56 | 9/8/2024 | 20°41′50″N, 104°40′59″E | 800 | 1 | Harp |  | NTS.2024.168 | *Hipposideros poutensis* | F | lactating |  |
| 57 | 9/8/2024 | 20°41′50″N, 104°40′59″E | 800 | 2 | Harp |  | NTS.2024.189 | *Hipposideros poutensis* | F | non-reproductive |  |
| 58 | 9/8/2024 | 20°41′50″N, 104°40′59″E | 800 | 2 | Harp |  | NTS.2024.191 | *Hipposideros poutensis* | F | non-reproductive |  |
| 59 | 9/8/2024 | 20°41′50″N, 104°40′59″E | 800 | 2 | Harp |  | NTS.2024.192 | *Hipposideros poutensis* | F | non-reproductive |  |
| 60 | 9/8/2024 | 20°42′54″N, 104°41′17″E | 750 | 2 | Harp |  | NTS.2024.172 | *Hipposideros poutensis* | F | non-reproductive |  |
| 61 | 9/8/2024 | 20°40′28″N, 104°41′07″E | 750 | 2 | Mist net |  | NTS.2024.176 | *Hipposideros poutensis* | M | unknown |  |
| 62 | 9/8/2024 | 20°40′28″N, 104°41′07″E | 750 | 2 | Mist net |  | NTS.2024.178 | *Hipposideros poutensis* | M | mature |  |
| 63 | 9/8/2024 | 20°42′54″N, 104°41′17″E | 750 | 2 | Harp | ✓ | NTS.2024.170 | *Hipposideros poutensis* | M | mature |  |
| 64 | 9/8/2024 | 20°42′54″N, 104°41′17″E | 750 | 2 | Harp |  | NTS.2024.171 | *Hipposideros poutensis* | M | unknown |  |
| 65 | 9/8/2024 | 20°42′54″N, 104°41′17″E | 750 | 2 | Harp |  | NTS.2024.180 | *Hipposideros poutensis* | M | unknown |  |
| 66 | 9/8/2024 | 20°42′54″N, 104°41′17″E | 750 | 2 | Harp |  | NTS.2024.181 | *Hipposideros poutensis* | M | unknown |  |
| 67 | 9/8/2024 | 20°42′54″N, 104°41′17″E | 750 | 2 | Harp |  | NTS.2024.182 | *Hipposideros poutensis* | M | unknown |  |
| 68 | 9/8/2024 | 20°42′54″N, 104°41′17″E | 750 | 2 | Harp |  | NTS.2024.184 | *Hipposideros poutensis* | M | unknown |  |
| 69 | 9/8/2024 | 20°41′18″N, 104°40′19″E | 950 | 2 | Mist net |  | NTS.2024.186 | *Myotis muricola* | F | non-reproductive |  |
| 70 | 9/8/2024 | 20°41′50″N, 104°40′59″E | 800 | 2 | Mist net |  | NTS.2024.185 | *Pipitrellus tenuis* | M | unknown |  |
| 71 | 9/8/2024 | 20°40′28″N, 104°41′07″E | 750 | 2 | Mist net |  | NTS.2024.177 | *Rhinolophus affinis* | F | non-reproductive |  |
| 72 | 9/8/2024 | 20°39′58″N, 104°40′55″E | 800 | 2 | Harp |  | NTS.2024.169 | *Rhinolophus pearsonii* | M | unknown |  |
| 73 | 9/8/2024 | 20°40′28″N, 104°41′07″E | 750 | 2 | Mist net |  | NTS.2024.175 | *Rhinolophus pearsonii* | M | unknown |  |
| 74 | 9/8/2024 | 20°41′50″N, 104°40′59″E | 800 | 2 | Harp |  | NTS.2024.190 | *Rhinolophus pearsonii* | F | non-reproductive |  |
| 75 | 9/8/2024 | 20°42′54″N, 104°41′17″E | 750 | 2 | Harp |  | NTS.2024.173 | *Rhinolophus pearsonii* | F | non-reproductive |  |
| 76 | 9/8/2024 | 20°42′54″N, 104°41′17″E | 750 | 2 | Harp |  | NTS.2024.174 | *Rhinolophus pearsonii* | F | non-reproductive |  |
| 77 | 9/8/2024 | 20°42′54″N, 104°41′17″E | 750 | 2 | Harp |  | NTS.2024.183 | *Rhinolophus pearsonii* | F | non-reproductive |  |
| 78 | 9/8/2024 | 20°41′50″N, 104°40′59″E | 800 | 2 | Harp |  | NTS.2024.187 | *Rhinolophus thomasi* | F | non-reproductive |  |
| 79 | 9/8/2024 | 20°41′50″N, 104°40′59″E | 800 | 2 | Harp |  | NTS.2024.188 | *Rhinolophus thomasi* | M | unknown |  |
| 80 | 9/8/2024 | 20°41′50″N, 104°40′59″E | 800 | 2 | Harp |  | NTS.2024.193 | *Rhinolophus thomasi* | M | unknown |  |
| 81 | 11/8/2024 | 20°40′23″N, 104°39′16″E | 700 | 2 | Mist net |  | NTS.2024.195 | *Cynopterus sphinx* | M | unknown |  |
| 82 | 11/8/2024 | 20°40′23″N, 104°39′16″E | 700 | 2 | Mist net |  | NTS.2024.197 | *Cynopterus sphinx* | F | carrying juvenile |  |
| 83 | 11/8/2024 | 20°40′23″N, 104°39′16″E | 700 | 2 | Mist net |  | NTS.2024.199 | *Cynopterus sphinx* | F | non-reproductive |  |
| 84 | 11/8/2024 | 20°43′09″N, 104°42′32″E | 700 | 4 | Harp | ✓ | NTS.2024.201 | *Hipposideros poutensis* | F | non-reproductive |  |
| 85 | 11/8/2024 | 20°43′09″N, 104°42′32″E | 700 | 4 | Harp |  | NTS.2024.202 | *Hipposideros poutensis* | F | lactating |  |
| 86 | 11/8/2024 | 20°43′09″N, 104°42′32″E | 700 | 4 | Harp |  | NTS.2024.204 | *Hipposideros poutensis* | F | non-reproductive |  |
| 87 | 11/8/2024 | 20°43′09″N, 104°42′32″E | 700 | 4 | Harp |  | NTS.2024.206 | *Hipposideros poutensis* | F | non-reproductive |  |
| 88 | 11/8/2024 | 20°43′09″N, 104°42′32″E | 700 | 4 | Harp |  | NTS.2024.207 | *Hipposideros poutensis* | F | non-reproductive |  |
| 89 | 11/8/2024 | 20°43′09″N, 104°42′32″E | 700 | 4 | Harp |  | NTS.2024.200 | *Hipposideros poutensis* | M | unknown |  |
| 90 | 11/8/2024 | 20°43′09″N, 104°42′32″E | 700 | 4 | Harp |  | NTS.2024.205 | *Hipposideros poutensis* | M | unknown |  |
| 91 | 11/8/2024 | 20°42′28″N, 104°41′42″E | 700 | 4 | Harp |  | NTS.2024.208 | *Hipposideros poutensis* | M | unknown |  |
| 92 | 11/8/2024 | 20°40′23″N, 104°39′16″E | 700 | 2 | Mist net |  | NTS.2024.203 | *Megaerops niphanae* | F | non-reproductive |  |
| 93 | 11/8/2024 | 20°42′50″N, 104°41′02″E | 600 | 4 | Mist net |  | NTS.2024.196 | *Megaerops niphanae* | F | lactating |  |
| 94 | 11/8/2024 | 20°43′09″N, 104°42′32″E | 700 | 4 | Harp |  | NTS.2024.198 | *Rhinolophus pearsonii* | F | non-reproductive |  |
| 95 | 12/8/2024 | 20°41′29″N, 104°42′12″E | 650 | 2 | Harp |  | NTS.2024.214 | *Aselliscus stoliczkanus* | F | non-reproductive |  |
| 96 | 12/8/2024 | 20°40′23″N, 104°39′16″E | 700 | 2 | Mist net |  | NTS.2024.218 | *Cynopterus sphinx* | F | lactating |  |
| 97 | 12/8/2024 | 20°40′23″N, 104°39′16″E | 700 | 2 | Mist net |  | NTS.2024.220 | *Cynopterus sphinx* | F | mature |  |
| 98 | 12/8/2024 | 20°42′50″N, 104°41′02″E | 600 | 4 | Mist net |  | NTS.2024.215 | *Cynopterus sphinx* | M | mature |  |
| 99 | 12/8/2024 | 20°41′29″N, 104°42′12″E | 650 | 2 | Harp |  | NTS.2024.210 | *Hipposideros poutensis* | M | mature |  |
| 100 | 12/8/2024 | 20°41′29″N, 104°42′12″E | 650 | 2 | Harp |  | NTS.2024.211 | *Hipposideros poutensis* | M | mature |  |
| 101 | 12/8/2024 | 20°40′23″N, 104°39′16″E | 700 | 2 | Mist net |  | NTS.2024.217 | *Megaerops niphanae* | M | unknown |  |
| 102 | 12/8/2024 | 20°42′50″N, 104°41′02″E | 600 | 4 | Mist net |  | NTS.2024.216 | *Megaerops niphanae* | M | unknown |  |
| 103 | 12/8/2024 | 20°42′50″N, 104°41′02″E | 600 | 4 | Mist net |  | NTS.2024.219 | *Megaerops niphanae* | F | non-reproductive |  |
| 104 | 12/8/2024 | 20°41′29″N, 104°42′12″E | 650 | 2 | Harp | ✓ | NTS.2024.209 | *Rhinolophus pearsonii* | F | lactating |  |
| 105 | 12/8/2024 | 20°41′29″N, 104°42′12″E | 650 | 2 | Harp |  | NTS.2024.213 | *Rhinolophus pusillus* | M | mature |  |
| 106 | 12/8/2024 | 20°42′50″N, 104°41′02″E | 600 | 4 | Mist net | ✓ | NTS.2024.212 | *Tynonycteris tonkinensis* | M | mature |  |
| 107 | 13/08/2024 | 20°41′38″N, 104°42′11″E | 500 | 4 | Harp |  | NTS.2024.221 | *Rhinolophus pearsonii* | F | non-reproductive |  |
| 108 | 15/08/2024 | 20°40′36″N, 104°41′18″E | 910 | 3 | Mist net |  | NTS.2024 | *Hipposideros poutensis* | M | mature |  |
| 109 | 15/08/2024 | 20°40′36″N, 104°41′18″E | 910 | 3 | Mist net |  | NTS.2024.223 | *Hipposideros poutensis* | M | mature |  |
| 110 | 26/03/2010 | - | 900 | 2 | Mist net |  | XN.03 | *Kerivoula* cf. *dongduongana* | F | non-reproductive |  |
| 111 | 26/03/2010 | - | 900 | 2 | Mist net |  | XN.04 | *Kerivoula* cf. *dongduongana* | F | non-reproductive |  |
| 112 | 26/03/2010 | - | 900 | 2 | Mist net |  | XN.06 | *Kerivoula* cf. *dongduongana* | F | non-reproductive |  |
| 113 | 26/03/2010 | - | 900 | 2 | Mist net |  | XN.07 | *Kerivoula* cf. *dongduongana* | F | non-reproductive |  |
| 114 | 26/03/2010 | - | 800 | 2 | Mist net |  | IEBR-M-2947 | *Myotis alticraniatus* | F | unknown |  |
