# Supplementary material for: Study of bat diversity (Mammalia, Chiroptera) in Xuan Nha Nature Reserve, Son La Province, north-western Vietnam, based on integrative insights from morphology, genetics and echolocation data
Source: Biodivers Data J. 2025 Nov 4;13:e165516. doi: 10.3897/BDJ.13.e165516 (PMC12606075; doi:10.3897/BDJ.13.e165516)
Supplement: Supplementary material 2 — List of craniodental measurements used in this study [file bdj-13-e165516-s002.docx]

**Supplementary material 2.** List of craniodental measurements used in this study.

| No. | Character | Abbreviations |  |
| --- | --- | --- | --- |
| Cranium | | | |
| 1 | GTL | Greatest length of skull (from the front of the 1st upper incisor to the most projecting point of the occipital region). |  |
| 2 | CCL | Condylo-canine length (distance from the exoccipital condyle to the most anterior part of the canine). |  |
| 3 | CM^3^L | Maxillary toothrow length (distance from the front of upper canine to the back of the crown of the third molar). |  |
| 4 | CP^4^L | Upper canine-premolar length (from the front of the upper canine to the back of the crown of the last premolar). |  |
| 5 | P^4^M^3^L | Upper molariform toothrow length (from the posterior upper premolar to the last molar). |  |
| 6 | M^1^M^3^L | Upper molar crown length (from the front of the 1st upper molar to the last molar). |  |
| 7 | MAW | Mastoid width (greatest distance across the mastoid region). |  |
| 8 | BCH | Braincase height (from the basisphenoid at the level of the hamular processes to the highest part of the skull, including the sagittal crest, if present). |  |
| 9 | BB | Breadth of braincase at the posterior roots of zygomatic arches. |  |
| 10 | RW | Rostrum width (the greatest width across the supraorbital tubercles) |  |
| 11 | IOW | Interorbital width (least width of the interorbital constriction). |  |
| 12 | ZYW | Zygomatic width (greatest width of the skull across the zygomatic arches). |  |
| 13 | C^1^C^1^W | Width across the upper canines (greatest width across the outer borders of the upper canines). |  |
| 14 | M^3^M^3^W | Width across the upper molars (greatest width across the outer crowns of the last upper molars). |  |
| Mandible | | | |
| 15 | ML | Mandible length (distance from the anterior rim of the alveolus of the first lower incisor to the most posterior part of the condyle). |  |
| 16 | CPH | Least height of the coronoid process (distance from the tip of the coronoid process to the apex of the indentation on the inferior surface of the ramus adjacent to the angular process). |  |
| 17 | cm_3_L | Mandibular tooth row length (distance from the front of the lower canine to the back of the crown of the third lower molar). |  |
| 18 | cp_4_L | Lower canine-premolar length (distance from the front of the lower canine to the back of the crown of the posterior premolar). |  |
| 19 | p_4_m_3_L | Lower molariform toothrow length (Posterior lower premolar to the last lower molar length). |  |
| 20 | m_1_m_3_L | Lower molars crown length. |  |
